# A randomised, prospective and head-to-head comparison of [^68^Ga]Ga-PSMA-11 and [^18^F]PSMA-1007 for the detection of recurrent prostate cancer in PSMA-ligand PET/CT—Protocol design and rationale

**DOI:** 10.1371/journal.pone.0270269

**Published:** 2022-07-19

**Authors:** Ian Alberts, Lukas Bütikofer, Axel Rominger, Ali Afshar-Oromieh

**Affiliations:** 1 Department of Nuclear Medicine, Inselspital, Bern University Hospital, University of Bern, Bern, Switzerland; 2 CTU Bern, Mittelstrasse, Bern, Switzerland; Public Library of Science, UNITED KINGDOM

## Abstract

**Background:**

A number of radiopharmaceuticals are available for the detection of recurrent prostate cancer (rPC), but few comparative imaging trials have been performed comparing them. In particular, there are no prospective head-to-head comparisons of the recently introduced [^18^F]PSMA-1007 to the existing standard of care [^68^Ga]Ga-PSMA-11. The purpose of this trial is to establish the non-inferiority of the new radiopharmaceutical in terms of the rate of PET-positive findings and to obtain an intra-individual comparison of accuracy and radiopharmaceutical kinetics.

**Methods:**

In this cross-over trial we will randomise 100 individuals to receive either first a standard-of-care PET/CT using [^68^Ga]Ga-PSMA-11 followed by an additional [^18^F]PSMA-1007 PET/CT within 2 weeks, or vice-versa. Inclusion criteria include patients 18 years and older with biochemical recurrence of prostate cancer following radical prostatectomy, defined as two consecutive prostate specific antigen (PSA) levels > 0.2 ng/ml. Detection rate at the patient-based level is the primary end-point. Each scan will be interpreted by a panel of six independent and masked readers (three for [^68^Ga]Ga-PSMA-11 and three for [^18^F]PSMA-1007) which consensus majority in cases of discrepancy. To confirm the PET-positivity rate at a patient based level, follow up at 6 months following the first scan will be performed to a composite standard of truth. Secondary endpoints shall include an intra-individual comparison of radiopharmaceutical-kinetics, per-region detection rate and positive predictive value.

**Discussion:**

This is the first randomised prospective comparative imaging trial to compare the established [^68^Ga]Ga-PSMA-11 with [^18^F]PSMA-1007 and will determine whether the new radiopharmaceutical is non-inferior to the established standard-of-care in terms of patient-level detection rate.

**Clinical trial registration:**

Registered with and approved by the regional ethics authority #2020–02903 (submitted 09.12.2020, approval 16.12.2021) and the regulatory authority SwissMedic 2020DR2103. Registered with ClinicalTrials.gov Identifier NCT05079828 and additionally in a national language in the Swiss National Clinical Trials Portal (SNCTP).

## Introduction

### Background

Prostate cancer is the most commonly new diagnosed cancer in men and second leading cause of cancer-related death in the United States [1]. Despite timely initial therapy, biochemical recurrence of disease post radiotherapy or prostatectomy is common. Accurate staging of recurrent disease guides therapeutic decision-making [2]. Combined positron emission and computed tomographies (PET/CT) using radio-ligands to the prostate membrane specific antigen (PSMA) is now a well-established tool for the staging of biochemically recurrent prostate cancer and is endorsed by both National Comprehensive Cancer Network (NCCN) guidelines and European Association of Urology Guidelines (EUA). The first clinically routine PSMA-radio-ligand, [^68^Ga]Ga-PSMA-11, demonstrated superior diagnostic performance (in terms of detection rate) when compared to a previous generation choline-based radiopharmaceuticals [3]. More recently, [^18^F]-labelled radiopharmaceuticals have been introduced and have a number of theoretical advantages compared to ^68^Ga. For example, the radiolabel, ^18^F, is more easily available and this may improve cost efficacy [4] as well as theoretically improving image quality through lower positron energy and a longer half-life [5], although improvements in examination protocols [6], scanner technology [7] and cyclotron-produced ^68^Ga may limit some of the reported advantages of [^18^F]-labelled radiopharmaceuticals.

Whereas the European Urology Association (EUA) guidelines for the investigation of biochemical recurrence recommend the use of any “PSMA-PET” scan, the National Comprehensive Cancer Network (NCCN) specify (at the time of writing) only [^68^Ga]Ga-PSMA-11 and [^18^F]-piflufolastat (also known as PYALRIFY^®^ or DCFPyL). Despite being grouped under the umbrella term “PSMA-PET”, the various PSMA-radioligands have rarely been subject to any formal head-to-head test, with only small, anecdotal or retrospective studies at risk of bias available to guide the choice between radioligands [8]. A recently performed systematic review and networked meta-analysis of all comparative imaging data in recurrent prostate cancer by our own group suggest differences in diagnostic performance [8], meaning that they cannot necessarily be regarded interchangeable. Furthermore, while prospective trial data is available to support the use of both [^68^Ga]Ga-PSMA-11 [9] and [^18^F]-piflufolastat, this is not the case for [^18^F]PSMA-1007 and this systematic analysis was unable to find any published comparative imaging studies [^18^F]PSMA-1007 and [^68^Ga]Ga-PSMA-11 in rPC.

As a result, although [^18^F]PSMA-1007 is in widespread clinical use, there is a paucity of high-quality evidence to support this. Moreover, the debate as to which radiopharmaceutical is best suited to rPC is not settled, and concerns have been raised about the limited performance of [^18^F]PSMA-1007, particularly with high rates of unclear bone lesions with high radiopharmaceutical uptake which might mimic bone metastases [10, 11]. The lack of comparative imaging data for this widely used radiopharmaceutical is therefore an unmet need in evidence-based nuclear imaging of prostate cancer, which this study seeks to address.

### Rationale for study design and hypothesis

Diagnostic radiopharmaceuticals can be compared against a number of metrics. Recently published trial data for [^68^Ga]Ga-PSMA-11 compared to the previous standard of care [^18^F]-Fluciclovine established the superiority of the former compared to the latter in terms of detection rate, i.e. the proportion of patients with PET-positive findings, which is often taken to be a surrogate of the radiopharmaceutical’s sensitivity [3]. In a cohort of patients with known biochemical recurrence, there are no true negatives, making specificity analysis unfeasible. The diagnostic accuracy can be assessed by means of the PPV, or rate of false positive findings, but this challenging to compare with adequate statistical power in the setting of a head-to-head study, where only a small subset of patients with biochemical recurrence will subsequently have confirmatory clinical data. Moreover, lacking a coordinate system where lesions can be unambiguously compared between readers and correlated with postoperative findings (e.g. histology), a multi-reader analysis of the PPV is challenging. Retrospective data from our own group suggest a non-significantly higher detection rate for [^18^F]PSMA-1007 compared to [^68^Ga]Ga-PSMA-11 [4]. We therefore aim to establish the non-inferiority of this new radiopharmaceutical, [^18^F]PSMA-1007, with respect to the established standard of care [^68^Ga]Ga-PSMA-11 in terms of PET-positivity rate. The secondary objectives will explore the region-based positive predictive value, tumour visibility in terms of standardised uptake value (SUV), tumour to background ratio (TBR) and the inter-reader reliability for the two radiopharmaceuticals using these prospective data.

In contrast to standard static acquisitions, PET/CT is increasingly able to characterise the dynamics of radiopharmaceutical uptake, which might confer improved diagnostic performance. Reporting of the *in vivo* kinetic parameters of various PSMA-radiopharmaceuticals is incomplete. Whereas Ringheim et al. report the kinetic parameters *K*_1_ through *K*_4_ for [^68^Ga]Ga-PSMA-11 [12], Sachpekidis et al. report only *K*_1_ and *K*_*3*_ in their parametric imaging study of [^18^F]PSMA-1007 [13]. No comparative intra-individual data is available which might confirm which radiopharmaceutical has a higher affinity for the PSMA-target, as measured by the parametric parameter *K*_D_. Recent work by our own group has established the feasibility of whole-body parametric imaging using a state-of-the art extended field-of-view PET/CT system [14]. We will therefore obtain parametric imaging parameters as a secondary endpoint from a one-hour dynamic acquisition for the first ten patients included in this study, and which will be published in a separate exploratory analysis.

**Fig 1:**
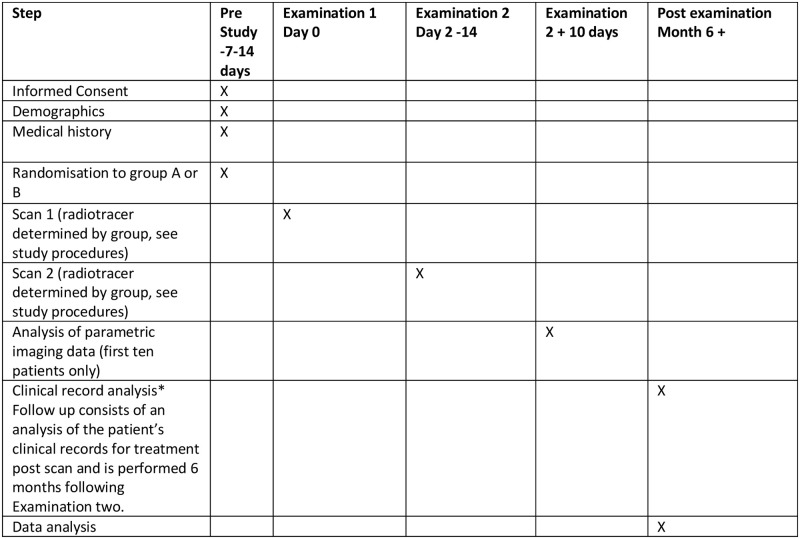
SPRIT schedule.

**Fig 2:**
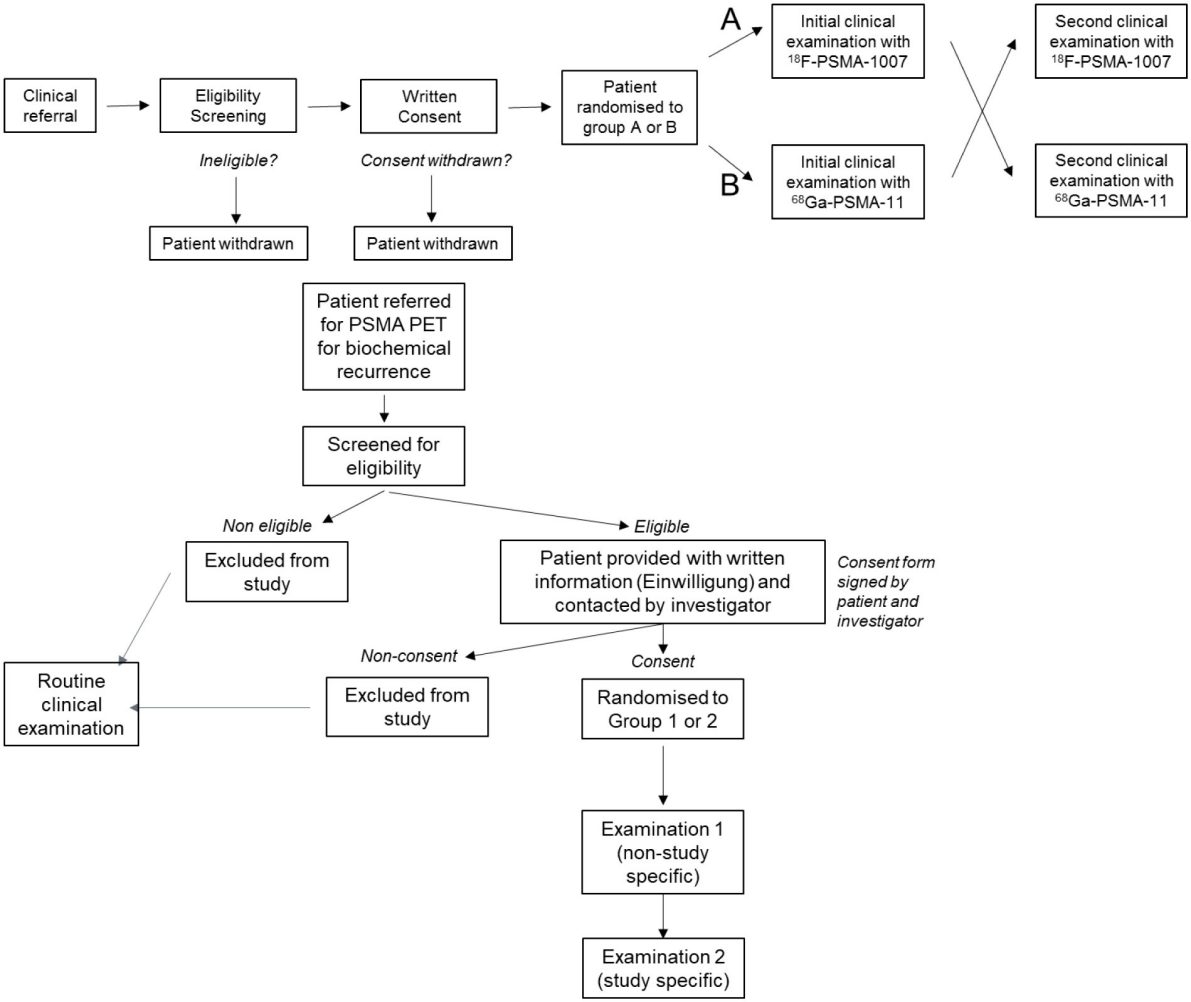
CONSORT study flow diagram.

The SPIRIT schedule of enrolment is shown in Fig 1 and CONSORT study flow chart is shown in Fig 2. All patients referred to our centre for PSMA-PET/CT for rPC will be screened for eligibility. Patients ineligible will receive a routine clinical examination and are excluded from the study. Those eligible will, once informed consent has been obtained, be randomised to one of two groups, who will receive PET/CT examinations using both radiopharmaceuticals using both radiopharmaceuticals in a cross-over fashion.

The study tests the null hypothesis that the new radiopharmaceutical is inferior to the old radiopharmaceutical with respect to the detection of patients with pathological PSMA-positive findings using a non-inferiority margin of -10% against the one-sided alternative that the new radiopharmaceutical is non-inferior to the old radiopharmaceutical.

### Objective of trial

The primary objective is to assess non-inferiority of the new radiopharmaceutical with respect to the proportion of patients with pathological PSMA-positive findings.

Secondary objectives are

- to compare the number of pathological, benign and uncertain lesions found with the two radiopharmaceuticals
- to determine the intra-reader agreement for the two radiopharmaceuticals
- to compare the proportion of pathological lesions confirmed in a follow-up between the two radiopharmaceuticals
- to compare tumour visibility between the two radiopharmaceuticals
- to evaluate safety and tolerability for the two radiopharmaceuticals
- to provide a head-to-head comparison of radiopharmaceutical kinetics in an exploratory analysis

#### Trial design

A prospective, single centre, open label, dual-arm, randomised, cross-over, pilot study comparing the new radiopharmaceutical [^18^F]PSMA-1007 with the standard radiopharmaceutical [^68^Ga]Ga-PSMA-11 in biochemically recurrent prostate cancer. This study will be conducted in compliance with the protocol, the current version of the Declaration of Helsinki, the international conference on harmonisation of technical requirements for registration of pharmaceutical for human use and good clinical practice (ICH-GCP) as well as all national legal and regulatory requirements. The protocol design takes into account the SPIRIT 2013 statement, and the SPIRIT checklist is provided as an appendix to this study.

#### Framework

Non-inferiority.

## Methods

### Study population

This single centre study will take place in the setting of a university hospital for nuclear medicine (Inselspital, Bern). The study population will include patients with biochemical recurrent of PC following primary radical prostatectomy (RPE). The recruitment target is n = 100 patients to afford appropriate statistical power for the primary endpoint. To allow a representative mix of patients that reflect clinical reality, we include patients with any PSA value (the only requirement is that they have a confirmed biochemical recurrence, i.e. PSA > 0.2 ng/ml post prostatectomy).

### Inclusion criteria

- Patients with known biochemical recurrence of a histologically confirmed prostate cancer post radical prostatectomy, defined as two consecutive PSA values > 0.2 ng/ml
- Patients > 18 y/o
- PSA measured within ± 4 weeks of the first PSMA-PET/CT
- Patients providing written informed consent
- No change in PC treatment in the period between the first and second scans

### Exclusion criteria

- Patients receiving ADT within 6 months prior to the PSMA-PET/CT
- Patients with contraindication to diuresis with 20mg Furosemide
- Patients with renal dialysis or relevant renal impairment (eGFR < 35 ml/min)
- Inability to provide written informed consent
- Inability to schedule and attend two consecutive PET examinations
- Patients undergoing active treatment for a second non-prostatic malignancy at the time of the first scan.
- Known sensitivity or allergy to PSMA-ligands or one of the components of the radiopharmaceutical solutions used.

### Intervention

In this cross-over trial, patients will be randomised to one of two radiopharmaceutical sequences, [^68^Ga]Ga-PSMA-11 first or [^18^F]PSMA-1007 first. Patients will undergo a scan with the first radiopharmaceutical, and a second scan at earliest after a period of 2 days and at latest 2 weeks. Under emergency circumstances, deviations from the protocol to protect the rights, safety and well-being of human subjects may proceed without prior approval of the sponsor and the competent authorities. Such deviations shall be documented and reported to the sponsor and the competent authorities as soon as possible.

### Quality assurance and control

Data will be entered directly using an electronic case report form (eCRF) and managed using a study specific REDCAP system. An external monitoring team from the Clinical Trials Unit (CTU) Bern monitors adherence to trial procedures. The monitoring plan uses a risk-based approach as published by the Swiss Clinical Trials Organisation (SCTO). The monitoring team are independent of the investigators and report directly to the sponsor.

### Study procedures

All patients will undergo scanning with both radiopharmaceuticals, affording an intra-patient comparison of both radiopharmaceutical types. The PET scans will be analysed with respect to number of lesions detected and radiopharmaceutical uptake (SUV) for pathological lesions.

### Investigational radiopharmaceutical

All patients will receive scans using both radiopharmaceuticals. In accordance with national prescribing guidelines, patients will receive 150 MBq ± 15% of [^68^Ga]Ga-PSMA-11 with imaging at 1h post injection of radiopharmaceutical (p.i.): Due to the urinary excretion exhibited by [^68^Ga]Ga-PSMA-11 which might obscure lesions contiguous with the urinary bladder [6], furosemide is given as per institutional protocol. 20mg of furosemide will be given intravenously (i.v.) 20–30 min prior to the PET scan [15]. Likewise, in accordance with national prescribing guidelines, patients will receive 300 MBq ± 15% of [^18^F]PSMA-1007 with imaging at 2h p.i. and where the lack of renal excretion makes additional preparations such as forced diuresis unnecessary.

### Source of the study drug

Both radiopharmaceuticals will be obtained from commercial radiopharmaceutical suppliers as per clinical routine and who operate in accordance with national guidelines.

### PET/CT imaging protocol and parametric imaging

PET/CT images will be acquired using the Siemens Biograph Quadra scanner at the University Hospital of Bern, Department of Nuclear Medicine in 3D mode, with time of acquisition in accordance with national prescribing guidance and as detailed above. Scan coverage will be from the vertex to the mid thighs. PET data will undergo attenuation correction and correction for scatter and randoms and using proprietary OSEM and PSF reconstruction. Acquisition time will be adapted to reflect the different dose and acquisition times for the radiopharmaceutical, to ensure comparable count-statistics when comparing lesion radiopharmaceutical uptake. For a subset of patients willing to undergo additional examination, (n = 10), images will be acquired for the first hour post injection of radiopharmaceutical in addition to the standard exam at 2h. From this, parametric (dynamic) data can be obtained, including (for the first time) an intra-individual comparison of radiopharmaceutical dynamics and ligand binding to tumours.

The parametric scans result in no additional radiation dose, except for an additional low-dose CT scan for attenuation correction (1mSv). The examination otherwise remains the same. After one hour, the patient waits as per clinical routine in the waiting area, and the clinically indicated scan is performed at the appointed time (2h p.i.).

### PET/CT imaging analysis

All images will be read by board-certified nuclear medicine physicians or dual-certified nuclear medicine physicians and radiologists. Readers will use appropriate software (Siemens Syngo.Via) and reporting monitors meeting appropriate national clinical guidelines. The trial physicians when reading the scans will be blinded to this report and the clinical details of the patient.

The details of the image analysis are as follows:

1. A total of 6 readers will be allocated to the study. 3 readers will be allocated to read all scans for one radiopharmaceutical, 3 readers will be allocated to read all scans for the second radiopharmaceutical. This ensures that both radiopharmaceuticals are interpreted separately, eliminating recall bias. The odd number of readers allows for simple-majority voting in case of disagreement (i.e. where one reader rates the scan as negative and two others positive). Readers will not be involved in routine clinical care of trial participants.
2. All readers will be experienced in reading scans with their respective radiopharmaceutical type (minimum 100 clinical scans). A training set of literature will be provided to all readers before the study commences, informing the reading physicians about potential pitfalls and normal distribution of both radiopharmaceutical types.
3. Visual analysis will be done according to previously published reading criteria (e.g. PSMA-RADS) to include pathological, non-pathological and diagnostically uncertain lesion types [16].
4. Reading will be done by the readers in a blinded, randomised fashion. The scans will be read at independent reading sessions. No reader will have access to the other readers’ results or interpretation.
5. Readers will report the number of lesions by lesion type (PSMA-RADS 1.0) and by region, as described above.
6. Both readers will record radiopharmaceutical uptake (SUV) and determine lesion to background ratio, which is defined as lesion SUV (peak) ÷ SUVmean of background reference region (defined by convention as the left gluteal musculature).
7. The principal investigator and his team will perform an unmasked scrutiny of medical records to 12-months post the second exam. Where available, a composite standard of correlative imaging, histology and prostate specific antigen will be compared affording a descriptive analysis of lesion-based diagnostic accuracy.

### PET/CT DICOM images

Images will be available to researchers using the institutional radiology information system (RIS) and picture archiving computer system (PACS) and delivered to external readers using anonymised data on CD or DVD.

### Outcome measures

#### Primary endpoint

The primary outcome is the proportion of patients with PSMA-positive pathological lesions (patient based sensitivity).

#### Secondary endpoints

- The number of PSMA-positive lesions defined as pathological, benign and uncertain per region
- Inter-reader reliability on the region level for benign, pathological and uncertain lesions.
- Region based positive predictive value (PPV) based on the subset of patients and lesions with follow-up data at 6 months
- Semi-quantitative tumour to background ratio (defined as lesion SUV ÷ SUV of reference background region, where the background uptake is defined by convention as the left gluteal musculature) will be summarised on a region-level
- Number and severity of adverse events up to 48h post exam.
- Parametric imaging will be performed in an exploratory analysis for the two radiopharmaceuticals in a subset of patients (n = 10)

#### Other endpoints of interest

In a second round of tumour classification, two readers (in consensus) will classify each lesion as pathological, benign or uncertain in a lesion-specific approach and perform a lesion-based follow-up to assess PPV on the lesion level.

### Screening and enrolment

Patients referred for clinically routine PSMA scanning for biochemically rPC cancer are screened for eligibility by an investigator. The study design calls for the patients to be randomised prior to the first examination. Therefore, following clinical referral, patients will be screened for eligibility. If eligible, patients are contacted and provided with the study information. If the patient agrees, following discussion with an investigator, to participate, a consent form will be provided for the patient to sign and return to us.

Patients are then randomised to group A or B and the first clinically indicated exam is scheduled. Should the patient refuse participation or withdraw his consent, the patient will receive the examination with one of the two radiopharmaceuticals according to availability. Such a routine examination could occur with either radiopharmaceutical, both of which are routinely used both at our centre and throughout Europe, and both of which are approved for use in Switzerland.

**Fig 3:**
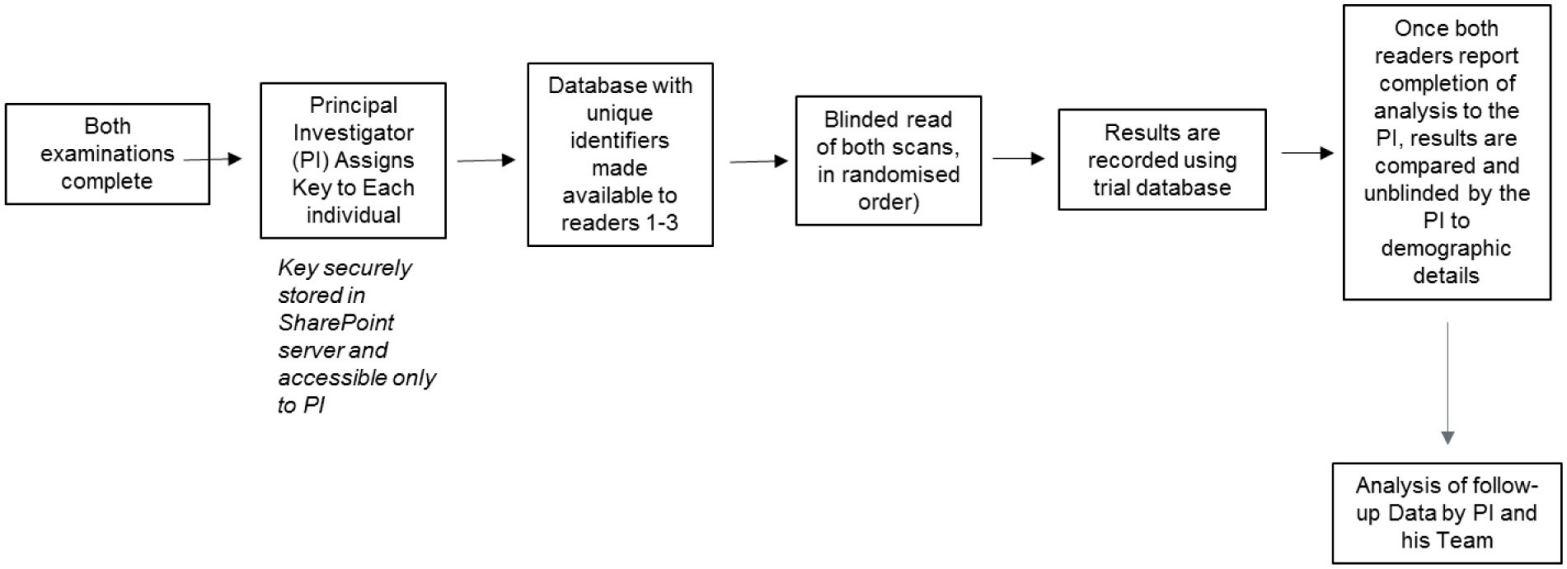
Study flow chart detailing consent procedures.

The patient will have time for reflection between receiving study information and receiving the first examination (min 24hrs). A second opportunity will occur to discuss the examination prior to the second (study-specific) examination. Consent will be obtained by means of a formal face-to-face meeting with a trial physician and the patient. The enrolment procedures are outlined in Fig 3.

### Randomisation and intervention

Patients will be randomised to one of two groups using a pragmatic approach with randomisation performed in REDCap. The investigators will have no way of predicting, or of influencing the group to which the individual is randomised.

The two groups are as followed:

A—Receive an [^18^F]PSMA-1007 scan initially, followed by [^68^Ga]Ga-PSMA-11
B—Receive a [^68^Ga]Ga-PSMA-11 scan initially, followed by [^18^F]PSMA-1007

### Follow-up

**Fig 4:**
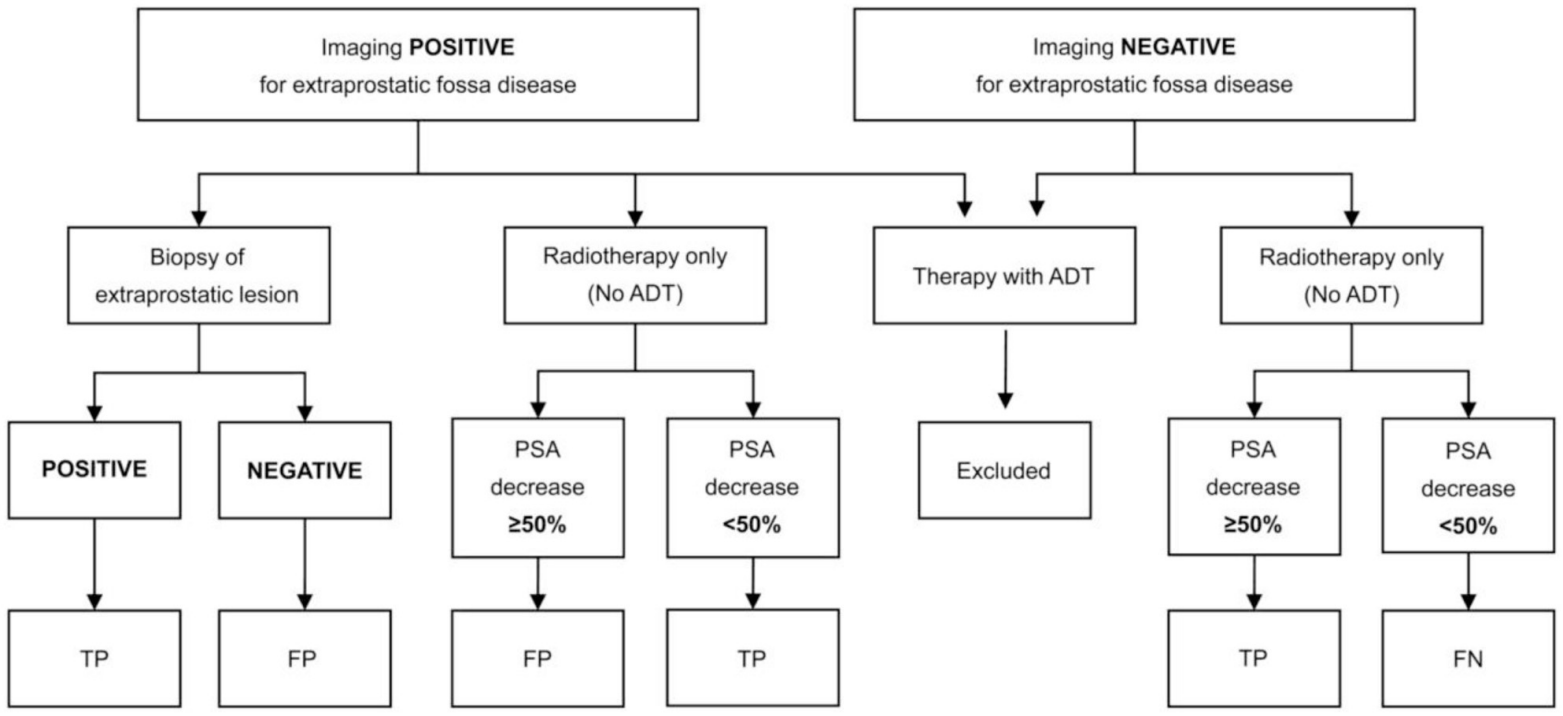
Example interpretation criteria for patients undergoing biopsy or radiotherapy (with and without systemic hormone deprivation therapy) image adapted from Emmett et al. [20].

Following the last PSMA-PET/CT, records will be scrutinised at twelve months of clinical follow up. Lesion validation will be performed using a composite standard of histology and/or correlative imaging as well as PSA-dynamics following external radiation therapy where available, affording a descriptive analysis of lesion-based diagnostic accuracy. Response to targeted treatment (surgery, radiotherapy) will be considered as confirmatory in the case of a fall in PSA, using previously published response criteria [17]. Where PET scan findings are recorded as positive, but the follow up refutes this, the scan will be recorded as false positive. Established RECIST (Vers 1.1) criteria will be used to interpret correlative imaging [18]. The details are as follows, and are outlined in Fig 4.

Follow up of clinical notes to 6 months will be performed for histopathological data, additional conventional imaging (CT, MRI and or bone scintigraphy), PSA change following focal salvage therapy (where no systemic therapy has been applied. Follow-up imaging will be performed by local read.

1. Follow up histology
  Positive biopsy or histology will be taken to be confirmatory.
  For negative biopsies: to rule out false negative imaging for image-guided biopsy will be reviewed. For lymph node biopsies, comparison of the PET/CT and follow-up (post intervention) imaging will be made to confirm that biopsy of the correct node occurred.
2. Follow up imaging
  No established imaging criteria are available for a number of imaging modalities in recurrent prostate cancer. To enable comparison of these data with previous imaging trials, imaging criteria as previously published by will be used [19].
3. Follow up following local treatment:
  PSA change following radiation therapy will be according to previously published criteria, with a PSA response of > 50% taken to be confirmatory [20] (see Fig 4).

### Study duration

We expect to enrol the 100 patients within 12 months of study initiation. Enrolment will remain open until 100 patients have received both the initial and study specific PET/CT examination.

### Sample size determination

Power was determined using Monte Carlo simulations with 1000 repetitions and the following assumptions:

- Marginal probabilities: 80% of the patients are positive for radiopharmaceutical A and 80% are positive for radiopharmaceutical B
- Both radiopharmaceuticals are applied to each patient
- Non-inferiority design with a one-sided alpha of 2.5%
- Non-inferiority margin of -10% (clinically acceptable margin in detection rate, balanced against the requirements for clinically realistic number of patients). We justify this margin as being less than 50% of the margin reported for superiority (22%) in a prospective trial published for [^68^Ga]Ga-PSMA-11 versus the first-generation radiopharmaceutical [^18^F]-Choline [17].
- Non-inferiority is concluded if the lower limit of a two-sided 95% confidence interval (Wald with Bonett–Price Laplace adjustment) for the difference in the proportion of radiopharmaceutical-positive patients (new radiopharmaceutical—old radiopharmaceutical) is larger than the non-inferiority margin
- A pragmatic assumption is made regarding the correlation of the paired measurements. Limited data are available for this, given the dearth of comparative studies. Neither retrospective analyses [21] nor limited pilot studies [22, 23] have demonstrated any difference in detection rate. We take a worst-case scenario, and assume that both radiopharmaceuticals are positive for 75% of the patients.

Data was simulated using function rmvbin from R package bindata [24]. With 100 patients this study will have a power of 85% to detect non-inferiority.

For the exploratory analysis of radiopharmaceutical kinetics (secondary endpoint), N = 10 patients is justified on the previous dosimetry paper for the established PET radiopharmaceutical [^68^Ga]Ga-PSMA-11 [25].

#### Strategies to ensure target recruitment

All patients referred for PSMA-PET/CT in rPC will be screened. We recognise that not all patients will meet our inclusion criteria or will be prepared to participate in this study, and the target sample size represents one fifth of all patients examined at our centre with PSMA-radiotracers yearly.

### Allocation sequence generation, concealment mechanism and implementation

Patients will be randomised to one of two groups using a pragmatic approach. A simple randomisation will be performed by the trial statistician using generation of a random number. The investigators will have no way of predicting, or of influencing the group to which the individual is randomised.

The two groups are as follows:

A—Receive an [^18^F]PSMA-1007 scan initially, followed by [^68^Ga]Ga-PSMA-11
B—Receive a [^68^Ga]Ga-PSMA-11 scan initially, followed by [^18^F]PSMA-1007

### Data collection, management and monitoring

Data is entered directly into an electronic Case Report Form (e-CRF). For each enrolled study participant an eCRF is maintained. eCRFs must be kept current to reflect subject status at each phase during the course of study. Participants will not be identified in the eCRF by name or initials and birth date. Appropriate coded identification by means of a unique participant number must be used. The principal investigator will maintain this list in the investigator site file and is only accessible to study team members but will be made available as appropriate during monitoring visits, audits and site inspections.

The eCRF will be made available to authorised personnel via the REDCap system. Only those authorised will have access to the eCRF. No investigator has access to another investigator’s eCRF. The Sponsor has access to all eCRFs.

Data collection will occur as follows:

1. Each individual patient will be assigned a unique patient identifier. Only the sponsor will have access to the “key”.
2. Imaging data will be coded and collected centrally by the sponsor. Coded and randomised data-sets will be provided to the readers at separate reading sessions. Data will be recorded separately on the CRF and the readers will not have access to each other’s results.
3. Once all data has been collected and the centralised reading is closed by the sponsor, the patient details will be unblinded to allow data analysis and clinical follow-up by the PI and his team of physician-investigators as named above.
4. The investigators will read each scan in a blinded fashion, and separately. The investigators will not have access to each other’s results.

The study specific read of the scans will occur separately to the local, documented clinical read with the report sent to the patients’ treating clinicians. No code break procedures apply to this study. Deviations from the original statistical analysis plan will be requested in writing by the PI to the trial statistician, and approved by the project sponsor.

External monitoring of data will be performed by the Clinical Trials Unit, Bern using a risk-based approach as published by the Swiss Clinical Trials Organisation, Bern.

### Statistical methods

The main analysis will include all randomised patients. Secondary analyses of all outcomes will be done in the per-protocol analysis set, excluding patients with major protocol deviations. No subgroup analyses are planned.

The primary outcome (proportion of patients with PSMA-positive pathological lesions) will be compared between radiopharmaceuticals using an absolute risk difference (new radiopharmaceutical minus old radiopharmaceutical) with a one-sided lower 95% confidence interval (CI) for paired proportions (Wald with Bonett–Price Laplace adjustment). If the lower limit lies above -10%, we will claim non-inferiority. Proportion of pathologic scans will be based on majority consensus reads.

The number of PSMA-positive lesions defined as pathological, benign and uncertain in each region will be analysed using separate mixed-effects Poisson regression models with radiopharmaceutical as fixed covariate and nested random effects for patient and region. We will report incidence rate ratios with 95% CI and p-values. As a sensitivity analysis we will compare the number over all regions using the Wilcoxon signed-rank tests and the Mann-Whitney statistic with 95% CI.

Inter-reader reliability will be analysed on the region-level based on the number of benign, pathological and uncertain lesions determined by each reader. We will calculate Krippendorff’s alpha with a ratio difference function and bootstrap 95% CI separately for each radiopharmaceutical.

Lesion-based PPV will be calculated for each radiopharmaceutical and will be presented with 95% Wilson score CI. Using all lesions with a confirmed status during follow-up, the two radiopharmaceuticals will be compared using McNemar’s test and an absolute risk difference with 95% confidence interval.

The semi-quantitative tumour to background ratio will be summarised on a region-level (i.e. using the mean over all positive lesions per region) and analysed using a linear mixed-effects regression model with radiopharmaceutical as fixed covariate and patient and region as nested random effects. The mean difference with 95% CI will be reported.

Parametric imaging will be performed for the two radiopharmaceuticals in a subset of patients (n = 10*), allowing comparison of the radiopharmaceuticals’ dosimetric and kinetic parameters, with affinity for the PSMA radioligand by calculation of the parametric equilibrium constant K_D_. These data will be reported in a separate exploratory analysis prior to analysis of the primary endpoint.

### Safety analyses

The number and severity of adverse events (up to 48h post exam) will be summarised by radiopharmaceutical using descriptive statistics. All serious adverse events will be listed.

### Interim analyses and handling of missing data

No interim analyses are planned. A separate exploratory analysis of the secondary endpoint (parametric imaging) will be performed prior to study completion. This analysis will be separate from the primary endpoint and will have no impact on the further process of the study. For patients lost to follow up, all reasonable measures to contact the patient or his treating physician will be taken. For patients who withdraw consent at any point, the data up to the time point of withdrawal will be used.

#### Protocol amendments

In this case that an amendment is required this will be submitted in writing to the sponsor and competent authorities.

#### Dissemination policy

The trial results will be published in a peer-reviewed journal and disseminated via national and international scientific congresses. Manuscript authorship will be in accordance with the principles set out in the ICJME (international committee of medical journal editors). As part of the peer-review process, access to the full trial protocol and coded data-sets may be provided at the discretion of the sponsor.

## Discussion

PET/CT using radioligands is a highly sensitive and accurate method for the detection of rPC. A large and increasingly bewildering array of PSMA-radioligands are available in the armamentarium of nuclear medicine physicians [26], including a number of novel ^18^F-labelled radioligands [5]. While some of these have been subject to comparative imaging trials compared to the previous standard of care [3], or analysed in prospective, Phase III diagnostic accuracy studies [9, 27], relatively fewer studies have been published for [^18^F]PSMA-1007, with retrospective reports [10, 28–30] or studies in small cohort without predefined power or hypothesis testing design [28]. An underpowered comparison in twelve patients with primary prostate cancer has been performed between [^18^F]DCFPyL and [^18^F]PSMA-1007, whose results are difficult to extrapolate to the rPC setting [31]. A previous systematic review from our own group of available comparative imaging data concluded that the lack of trials conducted with [^18^F]PSMA-1007 is an unmet need in evidence-based nuclear medicine. Whereas [^68^Ga]Ga-PSMA-11 and [^18^F]DCFPyL are explicitly mentioned in the NCCN guidelines, and have FDA approval, this is not the case for [^18^F]PSMA-1007, which in our own jurisdiction does not have marketing authorisation. Improved evidence may support the rationale for the use of this radiopharmaceutical [^18^F]PSMA-1007. Likewise, although not designed as a superiority trial or powered to test for diagnostic accuracy, by performing intra-individual comparison and with a reference standard of follow-up, should a clear advantage be identified for one radiopharmaceutical or a clear disadvantage for another in terms of diagnostic performance, we consider that these data would be of significant value for informing future decision making about which radiopharmaceuticals to implement for a rPC imaging programme.

A number of risks to this study can be identified. One risk is that additional trial data could be published before completion of this study. Only one other comparative trial in rPC could be identified in the ClinicalTrials.gov database (NCT04102553) which used ^18^F-Fluorocholine as the comparator or ^11^C-Choline (NCT03768349), neither of which use a current “gold-standard” PSMA-radioligand comparator. Should the required number of patients to reach statistical power (n = 100) fail to be obtained and the trial be terminated on grounds of failure to recruit, all planned analyses and follow up will nevertheless be performed to maximise the utility of the unique nature of the intra-individual comparative data obtained by this study. Further pitfalls could be loss of patients to follow-up. Supply issues for the radiopharmaceutical, particularly ^68^Ga-generators, may result in a longer than anticipated recruitment phase.

This is the first prospective randomised trial designed to determine the non-inferiority of [^18^F]PSMA-1007 compared to the standard [^68^Ga]Ga-PSMA-11 in terms of detection rate. Whereas published prospective comparative imaging trials are available for [^68^Ga]Ga-PSMA-11 [3] compared to other radiopharmaceuticals, this is not the case for [^18^F]PSMA-1007 [8].

## Supporting information

S1 File Clinical study protocol. (DOCX)
