# Supplementary material for: A randomised, prospective and head-to-head comparison of [68Ga]Ga-PSMA-11 and [18F]PSMA-1007 for the detection of recurrent prostate cancer in PSMA-ligand PET/CT—Protocol design and rationale
Source: PLoS One. 2022 Jul 19;17(7):e0270269. doi: 10.1371/journal.pone.0270269 (PMC9295986; doi:10.1371/journal.pone.0270269)
Supplement: S1 File — (DOCX) [file pone.0270269.s001.docx]

Clinical Study Protocol

Head-to-head comparison of ^68^Ga-PSMA-11 and ^18^F-PSMA-1007 for the detection of recurrent prostate cancer in PSMA-ligand PET/CT

A prospective, single centre, open label, dual-arm, randomised, cross-over, pilot study comparing the new tracer (^18^F-PSMA-1007) with the standard tracer (^68^Ga-PSMA-11) in biochemically recurrent prostate cancer.

| Study Type: | Clinical trial with Investigational Medicinal Product (IMP)- Radiopharmaceutical |
| --- | --- |
| Study Categorisation: | Risk category according to HRA: C |
| Study Registration: | NIH ClinicalTrials.gov NCT05079828 |
| Study Identifier: | 2020-02903 |
| Local Identifier: | “Gallium vs. fluorine PSMA” |
| Sponsor and Principal Investigator: | Sponsor: Prof. Dr. med. A. Rominger  Principal Investigator: Prof. Dr. med. A. Afshar-Oromieh  Klinik für Nuklearmedizin, Inselspital Bern  Freiburgstrasse 18  3010 Bern  Tel: 031-632-3655  Fax: 031-632-7663 |
| Investigational Product: | [(68)Ga]Ga-PSMA-11 and [(18)F]PSMA-1007 |
| Protocol Version and Date: | Version 2.1 24.03.2022 |

Study number 2020-02903

Study Title Head-to-head comparison of ^68^Ga-PSMA-11 and ^18^F-PSMA-1007 for the detection of recurrent prostate cancer in PSMA-ligand PET/CT.

The Sponsor and Principal Investigator have approved the protocol version [2.1, 24.03.2022], and confirm hereby to conduct the study according to the protocol, current version of the World Medical Association Declaration of Helsinki, ICH-GCP guidelines and the local legally applicable requirements.

Table of Contents

Study synopsis 6

Abbreviations 10

Study schedule 12

1.1.1 Study Calendar 12

1. STUDY ADMINISTRATIVE STRUCTURE 13

1.1 Sponsor 13

1.2 Principal Investigator(s) 13

1.3 Statistician ("Biostatistician") 13

1.4 Laboratory 13

1.5 Monitoring institution 13

1.6 Data Safety Monitoring Committee 13

1.7 Any other relevant Committee, Person, Organisation, Institution 13

2. ETHICAL AND REGULATORY ASPECTS 14

2.1 Study registration 14

2.2 Categorisation of study 14

2.3 Competent Ethics Committee (CEC) 14

2.4 Competent Authorities (CA) 14

2.5 Ethical Conduct of the Study 14

2.6 Declaration of interest 14

2.7 Patient Information and Informed Consent 14

2.7.1 Consent Procedure 15

2.8 Participant privacy and confidentiality 15

2.9 Early termination of the study 15

2.10 Protocol amendments 15

3. Background and Rationale 17

3.1 Background and Rationale 17

3.2 Investigational Product (treatment, device) and Indication 18

3.3 Preclinical Evidence 18

3.4 Clinical Evidence to Date 18

3.5 Dose Rationale 18

3.5.1 Parametric Imaging Protocol 19

3.6 Explanation for choice of comparator (or placebo) 19

3.7 Risks / Benefits 19

3.7.1 Risks 19

3.7.2 Benefits 19

3.8 Justification of choice of study population 20

4. STUDY OBJECTIVES 21

4.1 Overall Objective 21

4.2 Primary Objective 21

4.3 Secondary Objectives 21

4.4 Safety Objectives 21

5. STUDY OUTCOMES 22

5.1 Primary Outcome 22

5.2 Secondary Outcomes 22

5.3 Other Outcomes of Interest 22

5.4 Safety Outcomes 22

6. STUDY DESIGN 23

6.1 General study design and justification of design 23

6.1.1 Trial Design 23

6.1.2 Imaging Procedures 24

6.1.3 Follow up procedures 24

6.1.4 Response Criteria 24

6.2 Methods of minimising bias 25

6.2.1 Randomisation 25

6.2.2 Blinding procedures 25

6.2.3 Other methods of minimising bias 26

6.3 Unblinding Procedures (Code break) 26

7. STUDY POPULATION 27

7.1 Eligibility criteria 27

7.2 Recruitment and screening 27

7.3 Assignment to study groups 27

7.4 Criteria for withdrawal / discontinuation of participants 27

7.4.1 Data of withdrawn participants 27

7.4.2 Handling of Drop outs 27

8. STUDY INTERVENTION 28

8.1 Identity of Investigational Products (treatment / medical device) 28

8.1.1 Experimental Intervention (treatment / medical device) 28

8.1.2 Control Intervention (standard/routine/comparator treatment / medical device) 28

8.1.3 Packaging, Labelling and Supply (re-supply) 28

8.1.4 Storage Conditions 28

8.2 Administration of experimental and control interventions 28

8.2.1 Experimental Intervention 28

8.2.2 Control Intervention 28

8.3 Dose / Device modifications 28

8.4 Compliance with study intervention 28

8.5 Data Collection and Follow-up for withdrawn participants 28

8.6 Trial specific preventive measures 28

8.7 Concomitant Interventions (treatments) 29

8.8 Study Drug / Medical Device Accountability 29

8.9 Return or Destruction of Study Drug / Medical Device 29

9. STUDY ASSESSMENTS 30

9.1 Study flow chart(s) / table of study procedures and assessments 30

9.1.1 Study Procedures 30

9.1.2 Study Calendar 31

9.2 Assessments of outcomes 31

9.2.1 Assessment of primary outcome 31

9.2.2 Assessment of secondary outcomes 31

9.2.3 Assessment of other outcomes of interest 31

9.2.4 Assessment of safety outcomes 31

9.2.5 Assessments in participants who prematurely stop the study 31

9.3 Procedures at each visit 32

9.3.1 Initial (clinically routine, non-study specific) Examination 32

9.3.2 Second (study specific) Examination 32

10. SAFETY 33

10.1 Drug studies 33

10.1.1 Definition and assessment of (serious) adverse events and other safety related events 33

10.1.2 Reporting of serious adverse events (SAEs) and other safety related events 34

10.1.3 Follow up of (Serious) Adverse Events 34

10.2 Medical Device Category C studies 35

10.3 Medical Device Category A studies 35

10.4 Assessment, notification and reporting on the use of radiation sources 35

11. STATISTICAL METHODS 36

11.1 Hypothesis 36

11.2 Determination of Sample Size 36

11.3 Statistical criteria of termination of trial 36

11.4 Planned Analyses 36

11.4.1 Datasets to be analysed, analysis populations 36

11.4.2 Primary Analysis 36

11.4.3 Secondary Analyses 37

11.4.4 Subgroup analyses 37

11.4.5 Interim analyses 37

11.4.6 Safety analysis 37

11.4.7 Deviation(s) from the original statistical plan 37

11.5 Handling of missing data and drop-outs 37

12. QUALITY ASSURANCE AND CONTROL 38

12.1 Data handling and record keeping / archiving 38

12.1.1 Case Report Forms 38

12.1.2 Specification of source documents 38

12.1.3 Record keeping / archiving 38

12.2 Data management 38

12.2.1 Data Management System 38

12.2.2 Data security, access and back-up 38

12.2.3 Analysis and archiving 38

12.2.4 Electronic and central data validation 39

12.3 Monitoring 39

12.4 Audits and Inspections 39

12.5 Confidentiality, Data Protection 39

12.6 Storage of biological material and related health data 39

13. PUBLICATION AND DISSEMINATION POLICY 40

14. FUNDING AND SUPPORT 41

14.1 Funding 41

14.2 Other Support 41

15. INSURANCE 41

16. REFERENCES 42

17. APPENDICES 44

Study synopsis

Provide a structured synopsis containing all important information, preferably in tabular view:

| **Sponsor and Principal-Investigator** | Name of Sponsor: Prof. Dr. med. Axel Rominger  Name of Principal Investigator: Prof. Dr. med. Ali Afshar-Oromieh |
| --- | --- |
| **Study Title:** | Head-to-head comparison of ^68^Ga-PSMA-11 and ^18^F-PSMA-1007 for the detection of recurrent prostate cancer in PSMA-ligand PET/CT and determination of optimal imaging timing. |
| **Short Title / Study ID:** | 2020-02903 |
| **Protocol Version and Date:** | Version 2.1, 24.03.222 |
| **Trial registration:** | NIH ClinicalTrials.gov NCT 05079828 |
| **Study category and Rationale** | Category C: Study involving radiopharmaceuticals without approval in Switzerland |
| **Clinical Phase:** | Phase I/II study |
| **Background and Rationale:** | The aim of this study is to provide robust data on the head-to-head comparison of the two ligands of PSMA available in Switzerland for PET-imaging, i.e. ^68^Ga-PSMA-11 und ^18^F-PSMA-1007. |
| **Objective(s):** | The overall objective is to compare the performance of the new radiotracer for the investigation of prostate cancer (^18^F-PSMA-1007) in a head-to-head comparison with the existing standard-of-care (^68^Ga-PSMA-11).  The primary objective is to assess non-inferiority of the new tracer with respect to the proportion of patients with pathological PSMA-positive findings.  Secondary objectives are   - to provide a head-to-head comparison of tracer kinetics - to compare the number of pathological, benign and uncertain lesions found with the two tracers - to determine the intra-reader agreement for the two tracers - to compare the proportion of pathological lesions confirmed in a follow-up between the two tracers - to compare tumour visibility between the two tracers   to evaluate safety and tolerability for the two tracers |
| **Outcome(s):** | PRIMARY ENDPOINT  The primary endpoint is the proportion of patients with a pathological PSMA-positive finding (= positivity rate) at one time-point (2h) with the existing standard-of-care (^68^Ga-PSMA-11) compared to the new tracer (^18^F-PSMA-1007).  SECONDARY ENDPOINTS   - Intra-individual comparison of the parametric imaging parameter K*_D_* (measure of tracer affinity) for the two radiotracers. - The number of pathological, benign and uncertain lesions for each tracer in five regions (prostate bed, pelvic lymph nodes, extra-pelvic lymph nodes, bone, or other organs) classified by six blinded readers (three for each tracer) using previously published interpretation guidelines (PSMA-Rads 1.0) - Inter-reader reliability between the three readers for each tracer on the level of the region with respect to three categories (pathological, benign, uncertain). - The region-based positive predictive value (PPV), i.e. the percentage of pathological lesions that are confirmed pathological at the twelve months follow-up (based on the subset of patients and lesions with follow-up data at twelve months). - Tumour visibility will be compared by means of uptake values (SUV) as well as tumour to background contrast in a lesion based analysis. The tumour to background ratio (TBR) is defined as lesion SUV ÷ SUV of reference background region, where the background uptake is defined by convention as the left gluteal musculature. - Number and severity of adverse events per tracer (up to 48h follow up).   OTHER ENDPOINTS OF INTEREST  In a second round of tumour classification, two readers (in consensus) will classify each lesion as pathological, benign or uncertain in a lesion-specific approach and perform a lesion based follow-up to assess PPV on the lesion level. |
| **Study Design** | *A prospective, single centre, open label, dual arm randomised cross-over, non-inferiority trial comparing the new tracer (^18^F-PSMA-1007) with the standard tracer (^68^Ga-PSMA-11) in biochemically recurrent prostate cancer.*  *The study is designed using the Standards of Diagnostic Accuracy (STARD) checklist. The study is initiated, planned and will be conducted and analysed by the investigators. Financial support is provided the Werner und Hedy Berger-Janser Foundation, the Bernese Cancer league and the Direktion Lehre und Forschung, Inselspital Bern. The second tracer for the second PET is provided by either SWAN Isotopen AG or Unilabor Bern. All data will be collected in a central trial database.* |
| **Inclusion / Exclusion criteria:** | *Inclusion Criteria*   - Patients with known biochemical recurrence of a histologically confirmed prostate cancer post radical prostatectomy, defined as two consecutive PSA values > 0.2 ng/ml: - Post prostatectomy: Patients > 18 y/o - PSA measured within ± 4 weeks of the first PSMA-PET/CT - Patients providing written informed consent - No change in PC treatment in the period between the first and second scans   *Exclusion Criteria*   - Patients receiving ADT within 6 months prior to the PSMA-PET/CT - Patients with contraindication to diuresis with 20mg Furosemide - Patients with renal dialysis or relevant renal impairment (eGFR < 35 ml/min) - Inability to provide written informed consent - Inability to schedule and attend two consecutive PET examinations - Patients undergoing active treatment for a second non-prostatic malignancy at the time of the first scan. - Known sensitivity or allergy to PSMA-ligands or one of the components of the radiotracer solutions used. |
| **Measurements and procedures:** | All patients will undergo scanning with both radiotracers, affording an intra-patient comparison of both tracer types. The PET scans will be analysed with respect to number of lesions detected and tracer uptake (SUV) for pathological lesions. |
| **Study Product / Intervention:** | In this cross-over trial, patients will be randomised to one of two tracer sequences, ^68^Ga-PSMA-11 first or ^18^F-PSMA-1007 first. Patients will undergo a scan with the first radiopharmaceutical, and a second scan at earliest after a period of 2 days and at latest 2 weeks. |
| **Control Intervention (if applicable):** | Intra-patient comparison with both radiotracers. |
| **Number of Participants with Rationale:** | Recruitment will be open until 100 patients have been recruited.  Assuming that 80% of the patients are positive for each one of the tracers and that 75% of the patients will be positive for both tracers, recruitment of 100 patients will give a power of 85% to detect non-inferiority with a non-inferiority margin of -10% and a one-sided alpha of 2.5% using the Wald confidence interval for paired proportions with the Wald Bonett-Price Laplace adjustment. |
| **Study Duration:** | 2 years |
| **Study Schedule:** | First patient examination: June (planned) 2022*  Last patient examination: June 2023*  Follow-up of 6 months (until end of Dec 23)*  Data analysis and publication within 6 months (until June 2024)*  * See additional text in Section “Study Schedule” |
| **Investigator(s):** | Sponsor: Prof. Dr. med. Axel Rominger  Principal Investigator: Prof. Dr. med. Ali Afshar-Oromieh  Klinik für Nuklearmedizin, Inselspital Bern  Freiburgstrasse 18  3010 Bern  Tel: 031-632-3655  Fax: 031-632-7663 |
| **Study Centre(s):** | Single-centre (University Clinic for Nuclear Medicine, Inselspital Bern) |
| **Statistical Considerations:** | **Primary Endpoint:**  The primary outcome (proportion of patients with PSMA-positive pathological lesions = patient based sensitivity) will be compared between tracers using an absolute risk difference (new tracer minus old tracer) with a one-sided lower 95% confidence interval for paired proportions (Wald with Bonett–Price Laplace adjustment). If the lower limit lies above -10%, we will claim non-inferiority. Detection rates per patient will be based on majority consensus reads.  **Secondary Endpoints:**   - Parametric imaging will be performed for the two radiotracers in a subset of patients (n=10*), allowing comparison of the tracers’ dosimetric and kinetic parameters, with affinity for the PSMA radioligand by calculation of the parametric equilibrium constant *K*_D,_ which will be compared between the two tracers with descriptive statistics. - The number of PSMA-positive lesions defined as pathological, benign and uncertain per region will be compared using mixed-effects Poisson regression. Patient-level data will also be analysed using the Wilcoxon signed-rank test.. - Inter-reader reliability will be compared on the region level using Krippendorff’s alpha for benign, pathological and uncertain lesions, separately for the two tracers. - Region based PPV will be calculated for each tracer separately with 95% Wilson score confidence intervals. The two tracers will be compared using McNemar’s test and an absolute risk difference with 95% confidence interval. - Semi-quantitative tumour to background ratio (defined as lesion SUV ÷ SUV of reference background region, where the background uptake is defined by convention as the left gluteal musculature) will be summarised on a region-level (i.e. using the mean over all positive lesions per region). Summarised data will be analysed using linear mixed-effects regression models. - The number and severity of adverse event will be analysed using descriptive statistics. All serious adverse events will be listed.   *The requirement for N=10 patients is justified on the previous dosimetry paper for the established PET radiotracer ^68^Ga-PSMA-11 which was adequately powered to demonstrate a mean-effective dose of 0.023 ± 0.004 mSv/MBq, i.e. with a precision in the µSv range [1]. |
| **GCP Statement:** | This study will be conducted in compliance with the protocol, the current version of the Declaration of Helsinki, the ICH-GCP as well as all national legal and regulatory requirements. |

Abbreviations

| ADT | Androgen deprivation therapy |
| --- | --- |
| AE | Adverse Event |
| BASEC | Business Administration System for Ethical Committees, (https://submissions.swissethics.ch/en/) |
| CA | Competent Authority (e.g. Swissmedic) |
| CEC | Competent Ethics Committee |
| CRF | Case Report Form |
| ClinO | Ordinance on Clinical Trials in Human Research *(in German: KlinV, in French: OClin, in Italian: OSRUm)* |
| eCRF | Electronic Case Report Form |
| CTCAE | Common terminology criteria for adverse events |
| DSUR | Development safety update report |
| GCP | Good Clinical Practice |
| IB | Investigator’s Brochure |
| ICH | International conference on harmonisation of technical requirements for registration of pharmaceuticals for human use |
| Ho | Null hypothesis |
| H1 | Alternative hypothesis |
| HRA | Federal Act on Research involving Human Beings *(in German: HFG, in French: LRH, in Italian: LRUm)* |
| IMP | Investigational Medicinal Product |
| IIT | Investigator-initiated Trial |
| ISO | International Organisation for Standardisation |
| ITT | Intention to treat |
| MD | Medical Device |
| MedDO | Medical Device Ordinance *(in German: MepV, in French: ODim)* |
| PI | Principal Investigator |
| SDV | Source Data Verification |
| SOP | Standard Operating Procedure |
| SPC | Summary of product characteristics |
| SUSAR | Suspected Unexpected Serious Adverse Reaction |
| TMF | Trial Master File |

PC Prostate-Cancer

PET/CT Positron emission tomography/computed tomography

p.i. post injection of radiotracer

PPV Positive Predictive Value

PSMA Prostate specific membrane antigen

SUV standard uptake value

*SUVmax* = maximum SUV measured within a lesion

*SUVpeak =* defined as the average of a small, fixed region of the ROI (typically defined at 40% of the maximum) and is less vulnerable to image noise than SUVmax.

*SUVmean* = mean SUV measured within a volume of interest

StSV Strahlenschutzverordnung (814.501)

TBR tumour to background ratio

TNM International union against cancer classification of tumours (8^th^ Ed.)

ROI region of interest

VOI volume of interest

Study schedule


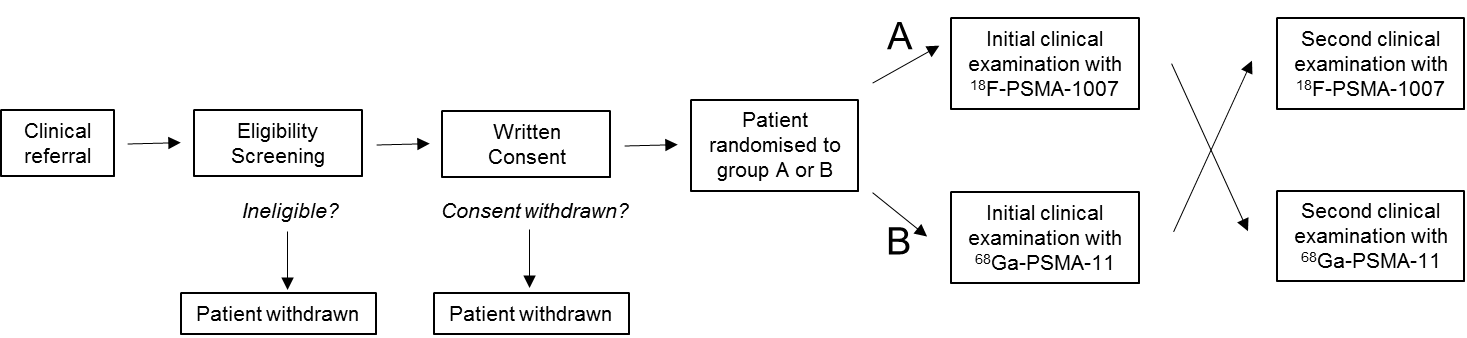


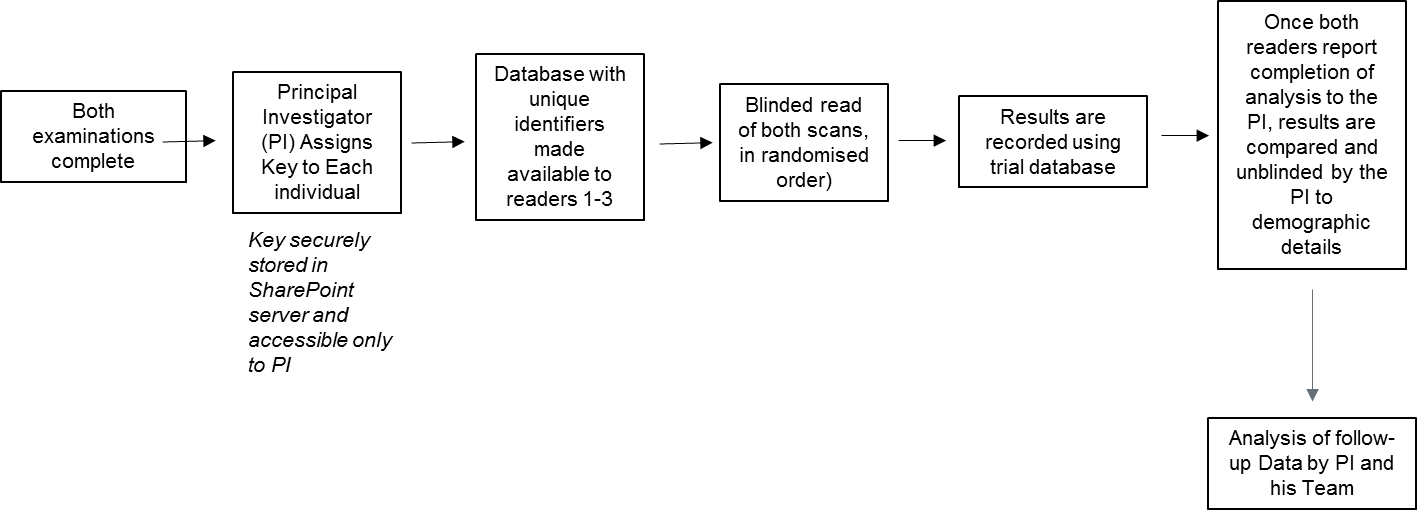


### Study Calendar

| **Step** | **Pre Study**  **-7-14 days** | **Examination 1**  **Day 0** | **Examination 2**  **Day 2 -14** | **Examination 2 + 10 days** | **Post examination**  **Month 6 +** |
| --- | --- | --- | --- | --- | --- |
| Informed Consent | X |  |  |  |  |
| Demographics | X |  |  |  |  |
| Medical history | X |  |  |  |  |
| Randomisation to group A or B | X |  |  |  |  |
| Scan 1 (radiotracer determined by group, see study procedures) |  | X |  |  |  |
| Scan 2 (radiotracer determined by group, see study procedures) |  |  | X |  |  |
| Analysis of parametric imaging data (first ten patients only) |  |  |  | X |  |
| Clinical record analysis*  Follow up consists of an analysis of the patient’s clinical records for treatment post scan and is performed 6 months following Examination two. |  |  |  |  | X |
| Data analysis |  |  |  |  | X |

# STUDY ADMINISTRATIVE STRUCTURE

## Sponsor

Prof. Dr. med. Axel Rominger

Klinik für Nuklearmedizin, Inselspital Bern

Freiburgstrasse 18

3010 Bern

Tel: 031-632-3655

Mail: axel.rominger@insel.ch

## Principal Investigator(s)

Prof. Dr. med. Ali Afshar-Oromieh

Klinik für Nuklearmedizin, Inselspital Bern

Freiburgstrasse 18

3010 Bern

Tel: 031-632-3655

Fax: 031-632-7663

Mail: ali.afshar@insel.ch

## Statistician ("Biostatistician")

Clinical Trials Unit Bern

Mittelstrasse 43

3012 Bern

Tel: 031 631 3372

www.ctu.unibe.ch

## Laboratory

No laboratory is associated with this trial.

## Monitoring institution

Monitoring will be carried out by a monitoring team from the clinical trials unit (CTU) Bern.

## Data Safety Monitoring Committee

Dr. med. I.L. Alberts along with the trial statistician will form a data safety monitoring committee and report to the PI any data safety issues. The committee is independent from the Sponsor and free from competing interests. The monitoring committee is supported by the trial study nurse..

## Any other relevant Committee, Person, Organisation, Institution

**Data Analysis Team:**

Dr. med. Ian L. Alberts, Prof. Dr. med. Ali Afshar-Oromieh

**Clinical Staff:**

Dr. med. Ian L. Alberts, Dr. med. Swantje Engelbrecht, Dr. med. Clemens Mingels, Dr. med. Dorothee Büsser, PD. Dr. T. Pyka., Dr. med. H. Rathke, Dr. med. L. Knappe, Dr. med. Gözlügöl, Dr. med. Akkurt

Radiographers/Technicians (dipl. Radiologie Fachfrau/Fachmann):

Mr Marco Viscione, Miss Angela Mendes, Mr Robin Schepers

# ETHICAL AND REGULATORY ASPECTS

Before the study is conducted, the protocol, the proposed patient information and consent form as well as other study-specific documents shall be submitted to a properly constituted Competent Ethics Committee (CEC) and/or competent authorities (Swissmedic and/or FOPH). The decision of the CEC, FOPH and Swissmedic concerning the conduct of the study will be made in writing to the Sponsor before commencement of this study. The clinical study can only begin once approval from all required authorities has been received. Any additional requirements imposed by the authorities shall be implemented.

## Study registration

The study is registered with ClinicalTrials.gov NCT05079828 (National Institutes of Health, USA). In addition, the study will be registered in a national language in the Swiss National Clinical Trials Portal (SNCTP via BASEC).

## Categorisation of study

The radiopharmaceuticals ^68^Ga-PSMA-11 and ^18^F-PSMA-1007 are authorised for use in Switzerland and used in accordance with the prescribed indication. The categorisation is Category C. We note the following:

1. The indication is within the same disease group of the International Classification of Diseases (ICD) (C61 Malignant neoplasm of prostate)

2. The authorised pharmaceuticals ^18^F-PSMA-1007 and ^68^Ga-PSMA-11 are used in accordance with the standard described in guidelines prepared in accordance with internationally accepted quality criteria (“Fachinformation”).

The clinically indicated scans will be performed using tracers which are both permitted for use in Switzerland by the regulatory authorities [2, 3] and will be used strictly in accordance with their prescribed indication. The total radiation dose per investigation is significantly below 20mSv.

## Competent Ethics Committee (CEC)

The sponsor and principal investigator are responsible for ensuring CEC authorisation prior to commencement of this study.

All changes in the research activity and all unanticipated problems involving risks to humans, including a planned or premature study end, will be reported, and the final report will be submitted to the appropriate committee. No changes will be made to the research protocol without prior CEC approval, except where necessary to eliminate apparent immediate hazards to study participants.

Premature study end or interruption of the study is reported within 15 days. The regular end of the study is reported to the CEC within 90 days, the final study report shall be submitted within one year after study end. Amendments are reported according to chapter 2.10.

## Competent Authorities (CA)

This application for a Category C study using radiopharmaceuticals will be submitted in parallel to Swissmedic and to the Federal Office of Public Health in accordance with §28 ClinO.

Reporting duties and timelines are the same as for the CEC, except for non-substantial amendments, which shall be reported as soon as possible. Amendments are reported according to chapter 2.10.

## Ethical Conduct of the Study

The study will be carried out in accordance to the protocol and with principles enunciated in the current version of the Declaration of Helsinki, the guidelines of Good Clinical Practice (GCP) issued by ICH, the Swiss Law and Swiss regulatory authority’s requirements. The CEC and regulatory authorities will receive annual safety and interim reports and be informed about study stop/end in agreement with local requirements.

## Declaration of interest

The sponsor, principal investigator and team declare no conflicts of interest.

## Patient Information and Informed Consent

The initial examination, irrespective of the tracer used, is performed as part of the routine treatment of the individual. Examinations with both tracers are performed many times per day at multiple centres in Switzerland. The initial examination, irrespective of tracer type, is reimbursed by the individual’s health insurance in accordance with Swiss health insurance regulations.

Before the initial investigation, the investigators will contact each individual by telephone to explain to each participant the nature of the study, its purpose, the procedures involved, the expected duration, the potential risks and benefits and any discomfort it may entail. Each participant will be informed that the participation in the study is voluntary and that he may withdraw from the study at any time and that withdrawal of consent will not affect his subsequent medical assistance and treatment.

The participant will be informed that his medical records may be examined by authorised individuals other than their treating physician.

All participants for the study will, in advance of informed consent being obtained by means of a face-to-face meeting (i.e. “Aufklärungsgespräch”), be provided with a participant information sheet and a consent form describing the study and providing sufficient information for participants to make an informed decision about their participation in the study. The information will be provided to the patients at least 24h in advance of being randomised to the first study. Patients can withdraw from the study at any time point and without prejudice to their treatment.

By adhering to this procedure, no delay will be incurred on the part of the patient in the scheduling of his examination, and a minimum of 24 hours is provided for the patient to consider whether he wishes to participate.

The formal consent of a participant, using the approved consent form, will be obtained before the participant is entered into the trial. The consent procedure will involve a face-to-face discussion with a medically qualified investigator. The form will be signed by the investigator “Prüfarzt” (or his designee) and patient.

The participant should read and consider the statement before signing and dating the informed consent form and should be given a copy of the signed document. The consent form must also be signed and dated by the investigator (or his designee), and it will be retained as part of the study records.

The patient can withdraw his consent at any time.

### Consent Procedure

Consent procedure is outlined in detail in §9.3

## Participant privacy and confidentiality

We affirm the individual's right to privacy, and the principal investigator will comply with applicable privacy laws. In particular, anonymity of the participants shall be guaranteed when presenting the data at scientific meetings or publishing them in scientific journals.

Individual subject medical information obtained as a result of this study is considered confidential and disclosure to third parties is prohibited. Subject confidentiality will be further ensured by utilising subject identification code numbers to correspond to treatment data in the computer files.

For data verification purposes, authorised representatives of the Sponsor, a competent authority (e.g. Swissmedic), or an ethics committee may require direct access to parts of the medical records relevant to the study, including participants’ medical history.

## Early termination of the study

The Sponsor (and any competent authority) may terminate the study prematurely under certain circumstances:

- Should the anticipated number of patients be reached earlier, then the study will be terminated.
- In the case of a recommendation by the data-safety monitoring committee.

The Sponsor may terminate the study prematurely under certain circumstances, for example:

- in case of ethical concerns,
- in case of insufficient participant recruitment,
- when the safety of the participants is doubtful or at risk, respectively,
- if alterations in accepted clinical practice make the continuation of a clinical trial unwise,
- in case of early evidence of benefit or harm of the experimental intervention

*Interruption of the study*

Occasionally, breaks in production of radiopharmaceuticals are experienced. In this case, should the delay or limited availability of the radiotracer impact upon the planned timing of the study, an amendment to the protocol with be sought for an equivalent period of time to the length of the interruption.

## Protocol amendments

The sponsor may amend the protocol with the approval of the sponsor, e.g. regarding changes to eligibility criteria, outcomes, analyses. Such amendments will be sent to all named parties in writing, and communicated to the CEC, competent authorities and trial registries. Substantial amendments are only implemented after approval of the CEC and CA, respectively.

Under emergency circumstances, deviations from the protocol to protect the rights, safety and well-being of human subjects may proceed without prior approval of the sponsor and the CEC/CA. Such deviations shall be documented and reported to the sponsor and the CEC/CA as soon as possible.

All non-substantial amendments are communicated to the CA as soon as possible, if applicable, and to the CEC with the Annual Safety Report (ASR).

# Background and Rationale

## Background and Rationale

Prostate Cancer (PC) is the most common malignancy in men and the second leading cause of cancer-related death in men [4]. Despite initial therapy at early stage disease, biochemical recurrence remains a commonly encountered entity and presents a challenge for conventional imaging modalities given their limited abilities to detect disease at early stages of recurrence (see figure 1) [5, 6]. An example of the difficulty posed by such patients in conventional imaging and the advantage of PET with PSMA-ligands is shown in figure 1.


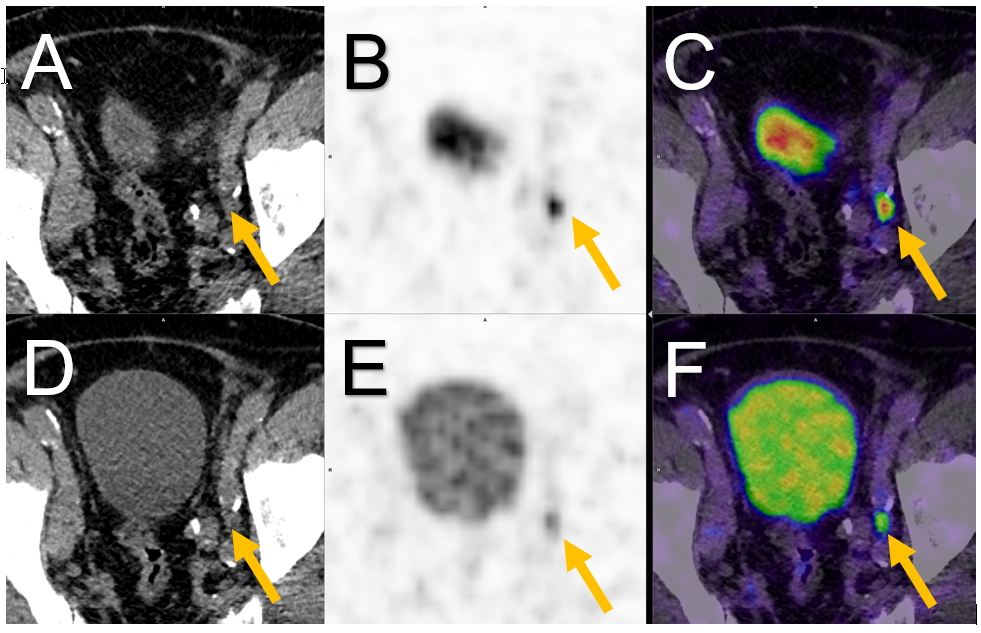


***Fig. 1.*** *Example PET/CT with ^68^Ga-PSMA-11* *in an individual with a lymph node metastasis (orange arrows). Tile A shows the low-dose CT (without contrast media), B shows the PET and C is a fusion of both, allowing for anatomic location of the increased uptake. Note that without the molecular imaging data provided by the PET, the lymph node is discernible only with great difficulty. Image courtesy of principal investigator [7].*

PET/CT with ligands of the prostate specific membrane antigen (PSMA) has been shown to have a significant impact on treatment and is now the *sine qua non* for staging of recurrent PC. For example, accurate identification of PC lesions allows for more accurate radiotherapy planning [8], allowing for an individualised treatment strategy. There is therefore a substantial clinical requirement for the accurate identification and stratification of individuals in whom prostate cancer is diagnosed [9] and at earlier stages of recurrent disease when the chance of a curative treatment is at its highest [10].

It is in this context that PSMA has become the focus of much attention owing to its high levels of expression on PC cells [6, 11] and has rapidly established itself as the investigation of choice in recurrent PC [5, 12-14]. Furthermore, PSMA-directed radioligand therapy is a rapidly evolving treatment modality for metastatic disease, creating an additional therapeutic role for PSMA-ligand molecular imaging, for which the term “theragnostics” has been coined [15]. The challenge for nuclear medicine is therefore to develop tracers and examination protocols that provide optimal detection and characterisation of disease, thus improving upon this promising technique.

The diagnostic PSMA-ligand ^68^Ga-PSMA-11, first introduced in 2011, has become the predominantly used PSMA-tracer in Switzerland. Although ^68^Ga-PSMA-11 reveals PC lesions often with excellent contrast, the short half-life of the isotope ^68^Ga (68min) creates handling difficulties and its excretion via the urinary system can complicate the diagnosis of local recurrences where bladder activity can obscure the prostate fossa. Recently, a novel PSMA-ligand has been introduced for PET-imaging (PSMA-1007) offering several potential advantages. Firstly, PSMA-1007 can be labelled with ^18^F, which has a longer half-life, thereby offering greater flexibility in the planning of scans. This longer half-life simplifies delivery of the radioisotope to peripheral clinics and the clinical routines involved in its use. Secondly, the physical properties of ^18^F potentially offer higher imaging resolution. Thirdly, unlike ^68^Ga-PSMA-11, ^18^F-PSMA-1007 often exhibits minimal excretion via the urinary bladder within the first 2-3h post injection (p.i.) tracer. This has the potential to improve the detection of local relapses adjacent to the urinary bladder wall.

There are currently no published prospective head-to-head studies comparing these two tracers in recurrent PC. These tracers are the only ones available in Switzerland. Because of this lack of data, there are no clear recommendations about which tracer to use and it which situation.

This study aims to fill this gap and provide comprehensive data with the potential to improve the diagnosis of PC. By providing robust data comparing the two tracers, such data will also provide guidance to clinicians faced with the scenario of an initially negative ^68^Ga-PSMA-11 PET as to the diagnostic utility of an additional ^18^F-PSMA-1007 PET, or vice-versa, and in which scenarios repeated scanning may be justified. In this regard, the impact of head-to-head data for these two tracers cannot be underestimated and is likely to yield significant influence on clinicians in their choice of PSMA-tracer and how they perform these scans. Such data would therefore have the potential to reduce the total radiation dose to patients by reducing unnecessary repeat scanning, and would also reduce costs to the health care system. This latter point is non-trivial considering prostate cancer’s high prevalence.

Finally, the application of the radiotracer into the same patient allows for a comparison of tracer kinetics. Although radiotracer kinetics are well known from the original pioneering dosimetric publications [1], they have never been compared in a head to head fashion. Obtaining dynamic scans over the first hour post injection will allow intra-individual dosimetry and a head-to-head comparison of parametric imaging parameters, allowing a direct comparison of the radiotracer’s affinity using standard parametric imaging techniques [16].

## Investigational Product (treatment, device) and Indication

We propose to investigate the two radiotracers ^68^Ga-PSMA-11 PET and ^18^F-PSMA-1007 PET. PSMA-radioligands (irrespective of type) are reimbursed indications for the examination of recurrent PC, defined as rising PSA post-treatment (§9.2(f), p. 106, Krankenpflege-Leistungsverordnung). Both radiopharmaceuticals are recognised for use in Switzerland by the regulatory authorities [2, 3]. Owing to the lack of “market authorisation” these must be regarded as investigational medical products, and therefore the study is categorised accordingly (Category C).

## Preclinical Evidence

Both tracers are authorised for use in Switzerland (as noted above, §3.2). For data regarding their first-use in humans, toxicology and dose data, we refer to the “Fachinformation” for the two substances.

## Clinical Evidence to Date

This investigation for these two radiopharmaceuticals will be the first such head-to-head comparison for this patient group. By definition, no systematic reviews are available. We are aware of no evidence of any reports of adverse safety profile, history of modification or recall of either of the substances.

We note one pilot study comparing these two radiotracers by Kuten et al., albeit in in a small cohort of primary prostate cancer patients and not in recurrent prostate cancer and in a study which did not conform to standard trial methodology [17]. Likewise, a retrospective comparison by Rauscher et al [13] exists, showing no inferiority between ^68^Ga-PSMA-11 PET and ^18^F-PSMA-1007 PET, although no sample size or power estimate was included. Both studies used a two point scale (binary positive/negative) and did not consider the possibility of diagnostic uncertainty, which is an under-investigated phenomenon and which this study seeks to address. A small case series reported increased diagnostic certainty for lesions in close proximity to the bladder with additional ^18^F-PSMA-1007, although we note the lack of diuretics for ^68^Ga-PSMA-11 as major weakness of this study [15]. This will be the first clinical study of prospective, randomised design conforming to standards for evidence-based medicine. The study design incorporates reduction of bladder activity through diuretic use, in-keeping with clinical evidence and guidelines. Diagnostic certainty in these tracers has hitherto been inadequately studied.

We note that PSMA radioligand uptake can be non-specific [16], and that non-prostatic tracer uptake is reported as increased in ^18^F-PSMA-1007 compared to ^68^Ga-PSMA-11 [13]. We consider this latter point to be of high clinical relevance: unnecessary additional diagnostic testing or treatment can result from diagnostically uncertain lesions.

## Dose Rationale

^68^Ga-PSMA-11 examination protocol

We will apply, in accordance with the “Fachliteratur”, 150 MBq ± 15% of ^68^Ga-PSMA-11.

Ideally, the dose applied would be the same as that of the comparator. As a result of a change in production of ^68^Ga-PSMA-11 from the Unilabor Bern to SWAN Isotopen AG, interruptions and periods of non-production are expected and have been experienced. In these cases, the radiopharmaceutical will be sourced from the Radiopharmazie of the USZ and from EURO-PET-GmbH (Freiburg im Breisgau, Germany). Here the radiopharmaceutical is produced under identical conditions, and both producers hold TLD licences. Higher doses (300 MBq) cannot be guaranteed as a result of the long logistics chain, and in any case, would be outwith the Fachliteratur. We will therefore apply 150 MBq but increase the acquisition time to obtain the same count statistics as if 300 MBq had been applied (this is equivalent to the photography concept of “exposure”). The “Fachliteratur” for ^18^F-PSMA-1007 defines, without any basis in evidence, an acquisition time of 2h; this is not specified in the literature for ^68^Ga-PSMA-11, although later acquisitions are known to lead to increased tumour-to-background ratios [18].

For all patients PET/CT examinations will be performed at 2h (± 10min) post application of the radiotracer as described, with image acquisition from the thighs to the skull base, as per institutional protocol. Before each scan, the patients will be asked to empty their bladder.

As described above, given the urinary excretion of ^68^Ga-PSMA-11 which can obscure lesions contiguous with the bladder, furosemide is given as per routine clinical protocol. 20mg of furosemide will be given intravenously (i.v.) 20-30 min prior to the final scan [19]. 1L of water will be provided to the patients to drink.

^18^F-PSMA-1007 examination protocol

We will inject a dose of 300 MBq ± 15% of ^18^F-PSMA-1007 intravenously with PET/CT examinations 2h post application of radiotracer as described in the “Fachinformation” (Appendix A) for ^18^F-PSMA-1007. PET/CT will be performed from thighs to skull base, as per institutional protocol. No further preparations are necessary due to the limited renal excretion of this tracer. The patient will empty his bladder prior to each scan.

### Parametric Imaging Protocol

For a subset of patients willing to undergo additional examination, (n=10), images will be acquired for ca. the first hour post injection of radiotracer in addition to the standard exam at 2h. Instead of the patient waiting on a chair for the hour(s) after injection, the radiotracer is applied while the patient lies on the scanner, which passively collects data regarding the tracer’s distribution in real-time. From this, parametric (dynamic) data can be obtained, including (for the first time) an intra-individual comparison of tracer dynamics and ligand binding to tumours.

The parametric scans result in no additional radiation dose, except for an additional low-dose CT scan for attenuation correction (1mSv). The examination otherwise remains the same. After one hour, the patient waits as per clinical routine in the waiting area, and the clinically indicated scan is performed at the appointed time (2h p.i.). A separate consent form detailing these extra procedures has been provided.

## Explanation for choice of comparator (or placebo)

A prospective, intra-patient comparison of the two tracers is required to compare their performance. This will provide generalisable data informing the choice of radiopharmaceuticals for prostate cancer imaging in future, and will benefit future patients. The data will provide an objective basis on which future decisions regarding tracer type and timing can be made by physicians.

## Risks / Benefits

### Risks

Given that the new tracer is now in routine clinical use in Switzerland and world-wide, no risk in terms of poor diagnostic performance can be anticipated from either radiotracer. Any additional radiation exposure entailed is within appropriate limits as defined by the FOPH (<20 mSv). The small risk of cancer induced by the radiation must be considered to be minimal for an aged population with a known cancer diagnosis undergoing examination for medical benefit.

Concerning the excellent safety profile of the radiopharmaceuticals, we draw particular attention to the relevant paragraph of the “Fachinformation”, for example for ^68^Ga-PSMA-PET/CT, which we quote here in the original German:

«Die ^68^Ga-PSMA-PET/CT ist mit einer geringen Strahlenexposition verbunden, die etwa der ein- bis zweifachen jährlichen natürlichen Strahlenexposition entspricht (ca. 3-6 mSv). Sie liegt unter der Strahlendosis der meisten konventionellen CT-Untersuchungen. Akute und/oder chronische Strahlenschäden durch ^68^Ga-PSMA-11 sind nicht zu erwarten oder je beobachtet worden. »

Furthermore, worldwide use of PSMA-ligands, including prospective phase II single arms trials (for ^68^Ga-PSMA-11 [20]), have reported no adverse safety events [25]. We highlight the fact that radiopharmaceuticals used as tracers are given in sub-pharmaceutical doses and no pharmaceutical effect is anticipated. The examination is routinely performed in men of advanced age with a known cancer diagnosis for medical benefit.

### Benefits

Although we cannot guarantee a clinical benefit to the individual by undergoing the additional examination, we note that a recently published initial case series suggests an increase in diagnostic certainty with additional ^18^F-PSMA-1007 PET/CT imaging [15] which may be of benefit to patients. All scans will be reported as per clinical routine, and this report will be forwarded to the patient’s treating physician, ensuring timely and accurate forwarding of relevant clinical information which may be obtained as a result of the imaging. The trial physicians will be blinded to these reports and any other clinical or demographic information when analysing the scans.

We expect that the information obtained from this study will be of benefit to future patients, through the provision of initial head-to-head data which will allow adequately powered trials to be done in the future.

**3.7.3 Threats to Study**

At the time of submission, no competing trials were registered in the EU Clinical Trials Register or the NIH Register. All funding is already available. No risks to participation are anticipated.

**3.7.4 Minimisation of Risk**

Verification procedures are in place (in accordance with GCP) for verification of the correct dose of the radiopharmaceutical and verification of the route of administration. Reporting of adverse events will be as outlined further in this protocol. The tolerability of the radiopharmaceutical will be recorded at the time of examination (interview by a physician prior to patient’s discharge from the clinic).

## Justification of choice of study population

Adult men suffering from prostate cancer and fulfilling our inclusion criteria. No children or vulnerable adults will be included in this study.

# STUDY OBJECTIVES

## Overall Objective

The overall objective is to compare the performance of the new radiotracer for the investigation of prostate cancer (^18^F-PSMA-1007) in a head-to-head comparison with the existing standard-of-care (^68^Ga-PSMA-11).

## Primary Objective

The primary objective is to assess non-inferiority of the new tracer with respect to the proportion of patients with pathological PSMA-positive findings.

## Secondary Objectives

Secondary objectives are

- to provide a head-to-head comparison of tracer kinetics

- to compare the number of pathological, benign and uncertain lesions found with the two tracers

- to determine the intra-reader agreement for the two tracers

- to compare the proportion of pathological lesions confirmed in a follow-up between the two racers

- to compare tumour visibility between the two tracers

- to evaluate safety and tolerability for the two tracers

## Safety Objectives

No adverse safety events are anticipated for these two routinely used radiopharmaceuticals. First-in-human studies have been published confirming the safety and dosage for both radiopharmaceuticals, which this study will not recapitulate. However, as a safety objective we plan to evaluate safety and tolerability for the two tracers up to 48h post-scan.

# STUDY OUTCOMES

## Primary Outcome

The primary endpoint is the proportion of patients with a pathological PSMA-positive finding (= detection rate) at one time-point (2h) with the existing standard-of-care (68Ga-PSMA-11) compared to the new tracer (^18^F-PSMA-1007).

## Secondary Outcomes

- Intra-individual comparison of the parametric imaging parameter K*_D_* (measure of tracer affinity) for the two radiotracers for n=10 patients.
- The number of pathological, benign and uncertain lesions for each tracer in five regions (prostate bed, pelvic lymph nodes, extra-pelvic lymph nodes, bone, or other organs) classified by six blinded readers (three for each tracer) using previously published interpretation guidelines (PSMA-Rads 1.0)
- Inter-reader reliability between the three readers for each tracer on the level of the region with respect to three categories (pathological, benign, uncertain).
- The region-based positive predictive value (PPV), i.e. the percentage of pathological lesions that are confirmed pathological at the 6 month follow-up (based on the subset of patients and lesions with follow-up data at 6 months).
- Tumour visibility will be compared by means of uptake values (SUV) as well as tumour to background contrast in a lesion based analysis. The tumour to background ratio (TBR) is defined as lesion SUV and SUV÷ SUV of reference background region, where the background uptake is defined by convention as the left gluteal musculature.
- Number and severity of adverse events per tracer (up to 48h follow-up, see below)

## Other Outcomes of Interest

In a second round of tumour classification, two readers (in consensus) will classify each lesion as pathological, benign or uncertain in a lesion-specific approach and perform a lesion based follow-up to assess PPV on the lesion level. This would be impossible to determine using a non-consensus multi-reader approach: lacking a coordinate system for the human body which could unequivocally describe each lesion.

## Safety Outcomes

We do not anticipate any adverse events. No known mechanism for anaphylaxis is known. However, in the unlikely case of an adverse event type (codified according to MedDRA) and severity of side effects will be recorded. Each patient receiving a scan will be evaluated for safety. The patient will be interviewed before and after the scan and spontaneous reports of adverse events reported to the investigator by patients and at the 48h follow up with the study nurse.

# STUDY DESIGN

## General study design and justification of design

Given the complexity of conducting nuclear imaging procedures and the multiple factors that must be borne in mind when attempting a fair and scientific comparison of the two substances, we outline the reasoning behind our numerous considerations here.

It is known that androgen deprivation therapy (ADT), a commonly prescribed treatment in recurrent prostate cancer, can affect PSMA expression of castration sensitive PC lesions in PSMA-PET/CT [27], resulting in unpredictability of the PET results. We therefore exclude patients undergoing ADT 6 months prior to the scans.

Forced diuresis is recommended in ^68^Ga-PSMA-PET/CT [17] due to high levels of tracer accumulation in the bladder, which potentially obscures lesions adjacent to the bladder, as shown in figure 3. This also facilitates a fair comparison between ^68^Ga-PSMA-PET/CT and ^18^F-PSMA-1007, which as outlined above, does not exhibit relevant urinary excretion within the first 2-3 hours p.i. [28]. Administration of intravenous (i.v.) furosemide (20 mg 30min before the final scan) and oral hydration (1L) is therefore included in the protocol for ^68^Ga-PSMA-11, and has been shown to reduce urinary bladder activity, which improves the visualisation of lesions adjacent thereto (see figure 3).

Identifying the optimal time-point at which to administer hydration and diuretics is hampered by the lack of data regarding the optimal imaging protocol. Forced diuresis too early following injection of the radiotracer is to be discouraged due to reduction of tracer availability at later time-points (2h) [22]. Given that increasing uptake over time is to be expected for PC lesions [23, 29], we favour application of diuretics as late as reasonably practical. In contrast, ^18^F-PSMA-1007 does not exhibit relevant urinary excretion within the first 2-3 hours after injection, thus making application of furosemide unnecessary [28].

Acquisition of imaging at time-points less than 1h p.i. is not recommended in PSMA-ligand PET due to the limited tracer uptake by PC lesions [21, 22], which is known to increase with time for PSMA-ligands [22, 23]. This pharmacokinetic property favours later acquisition, particularly in cases of diagnostic uncertainty [29]. However, we consider the acquisition of images at time-points later than 2h to be impractical due to the short half-lives of the radiopharmaceuticals which requires much larger initial doses of the radiopharmaceutical or significantly longer acquisition times [17]. As such, we compare both tracers at an equivalent time point, with 2h chosen. Given that the radiotracer will be administered only once, and a low dose CT is used for attenuation correction, our proposed protocol ensures that the total radiation exposure for the individual is kept to the lowest practicable level.

***Fig 3.*** *A lesion in the superior prostatic fossa is visible in the bottom row (post forced diuresis, 68Ga-PSMA-11 PET/CT), but indistinguishable from the bladder activity in the top row (without diuresis) in the same patient. Image courtesy of principal investigator*.

### Trial Design

The trial is designed using the Standards for Reporting of Diagnostic Accuracy Studies 2015 guidelines. This prospective, randomised, cross-over trial aims to provide a fair comparison between the two tracers in order to establish non-inferiority of the new tracer (^18^F-PSMA-1007) compared to the standard of care in Switzerland (^68^Ga-PSMA-11).

The trial is of open label design, insofar as the investigators will know which radiotracer was used: the normal biodistribution of the two tracers is different, for example, as described above ^68^Ga-PSMA-11 undergoes renal excretion, whereas ^18^F-PSMA-1007 shows little relevant renal excretion in the first two hours. Any trained nuclear medicine physician will be able to recognise the tracer-type immediately. A closed label design is therefore not practicable.

#### Design

The study is designed as a non-inferiority study with n=100 patients. To allow a representative mix of patients that reflect clinical reality, we include patients with any PSA value (the only requirement is that they have a confirmed biochemical recurrence, i.e. PSA > 0.2 ng/ml post prostatectomy).

### Imaging Procedures

Imaging will be performed on the same scanner for all patients at the clinic for nuclear medicine, university hospital Bern. PET scans will be obtained using standard parameters for the scanner type. All examinations will be documented as per clinical routine. The trial physicians when reading the scans will be blinded to this report and the clinical details of the patient.

*6.1.2.1 Imaging Protocol*

Patients will receive standard PET/CT acquisitions as per institutional imaging protocol at 2h p.i. For n=10 individuals, additional dynamic imaging will be performed from the time-point of injection to 80 min (dynamic scans)

*6.1.2.2 Details of analysis*

1. A total of 6 readers will be allocated to the study. 3 readers will be allocated to read all scans for one tracer, 3 readers will be allocated to read all scans for the second tracer. This ensures that both radiotracers are interpreted separately. The odd number of readers allows for simple-majority voting in case of disagreement (i.e. where one reader rates the scan as negative and two others positive).
2. All readers will be experienced in reading scans with their respective tracer type (minimum 100 clinical scans). A training set of literature will be provided to all readers before the study commences, informing the reading physicians about potential pitfalls and normal distribution of both tracer types.
3. Visual analysis will be done according to previously published reading criteria (e.g. PSMA-RADS) to include pathological, non-pathological and diagnostically uncertain lesion types [21].
4. Reading will be done by the readers in a blinded, randomised fashion. The scans will be read at independent reading sessions. No reader will have access to the other readers’ results or interpretation.
5. Readers will report the number of lesions by lesion type (PSMA-RADS 1.0) and by region, as described above.
6. Both readers will record tracer uptake (SUV) and determine lesion to background ratio, which is defined as lesion SUV (peak) ÷ SUVmean of background reference region (defined by convention as the left gluteal musculature).
7. The principal investigator and his team will perform an unmasked scrutiny of medical records to 6-months post the second exam. Where available, a composite standard of correlative imaging, histology and prostate specific antigen will be compared affording a descriptive analysis of lesion-based diagnostic accuracy.

#### Clinical Procedures

Both scans will be reported as per clinical routine. The scans are provided separate to the clinical trial by physicians not involved in the trial (i.e. none of the 6 readers will be involved in routine clinical care of the patients). In this way, the clinical treatment of the patient will not be adversely affected or delayed by participation in the trial.

### Follow up procedures

Following the last PSMA-PET/CT, records will be scrutinised at twelve months of clinical follow up. Lesion validation will be performed using a composite standard of histology and/or correlative imaging as well as PSA-dynamics following external radiation therapy where available, affording a descriptive analysis of lesion-based diagnostic accuracy.

### Response Criteria

Response to targeted treatment (surgery, radiotherapy) will be considered as confirmatory in the case of a fall in PSA, using previously published response criteria [22]. Where PET scan findings are recorded as positive, but the follow up refutes this, the scan will be recorded as false positive. Established RECIST (Vers 1.1) criteria will be used to interpret correlative imaging [23]. The details are as follows:

Follow up of clinical notes to 6 months will be performed for histopathological data, additional conventional imaging (CT, MRI and or bone scintigraphy), PSA change following focal salvage therapy (where no systemic therapy has been applied. Follow-up imaging will be performed by local read.

1. **Follow up histology**

Positive biopsy or histology will be taken to be confirmatory.

For negative biopsies: to rule out false negative imaging for image-guided biopsy will be reviewed. For lymph node biopsies, comparison of the PET/CT and follow-up (post intervention) imaging will be made to confirm that biopsy of the correct node occurred.

1. **Follow up imaging**

No established imaging criteria are available for a number of imaging modalities in recurrent prostate cancer. To enable comparison of these data with previous imaging trials, the imaging criteria as previously published by Fendler et al. will be used (§2.1.5.3 of Supplementary Materials 2: Trial Protocol [24])

1. **Follow up following local treatment**:

PSA change following radiation therapy will be according to previously published criteria, with a PSA response of > 50% taken to be confirmatory [25] (see figure 4).


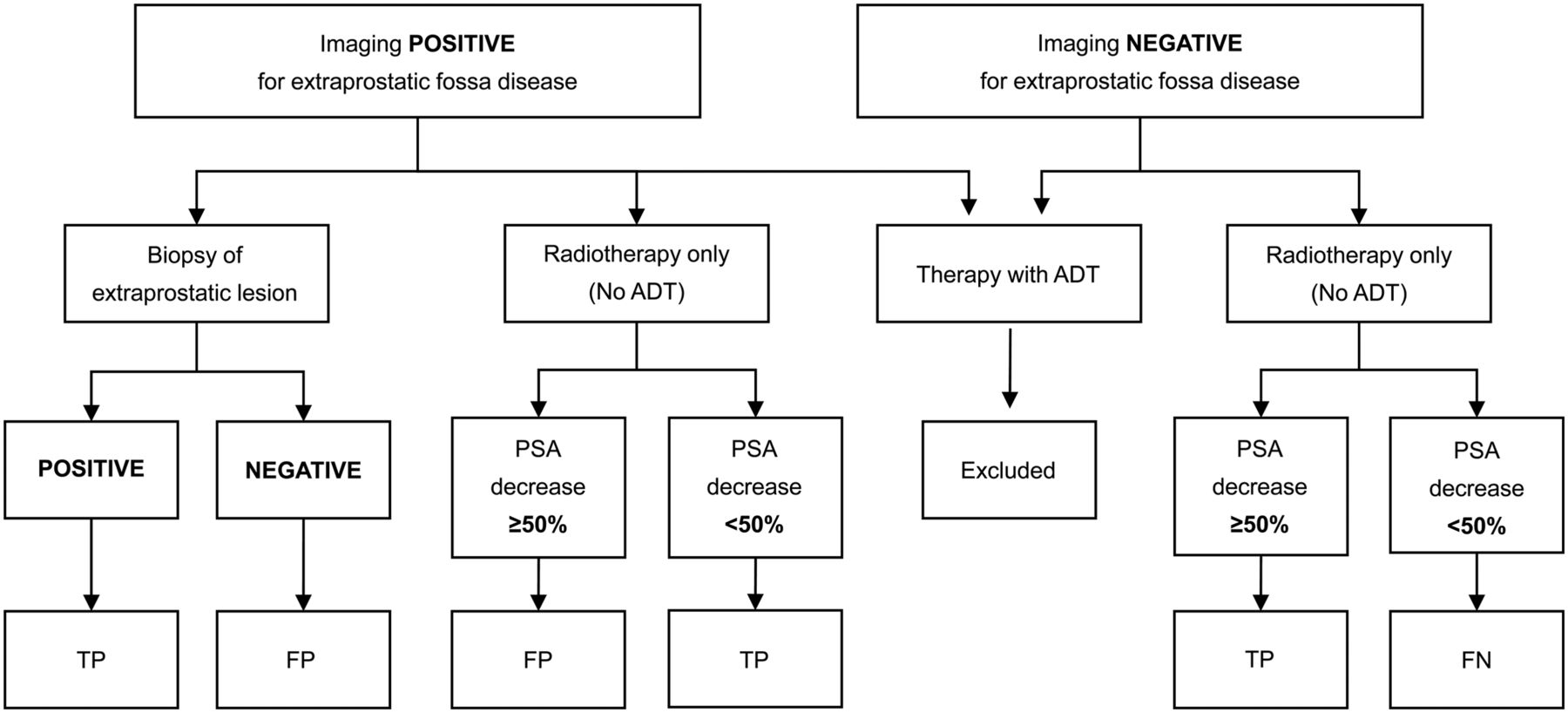


**Fig.4.** Example interpretation criteria for patients undergoing biopsy or radiotherapy (with and without systemic hormone deprivation therapy) image from Emmett et al. [25]

## Methods of minimising bias

### Randomisation

Patients will be randomised to one of two groups using a pragmatic approach. A simple randomisation will be performed by the trial statistician using generation of a random number. The investigators will have no way of predicting, or of influencing the group to which the individual is randomised.

The two groups are as followed:

A – Receive an ^18^F-PSMA-1007 scan initially, followed by ^68^Ga-PSMA-11

B – Receive a ^68^Ga-PSMA-11 scan initially, followed by ^18^F-PSMA-1007

### Blinding procedures

Data collection will occur as follows:

1. Each individual patient will be assigned a unique patient identifier. Only the sponsor will have access to the “key”.

2. Imaging data will be coded and collected centrally by the sponsor. Coded and randomised data-sets will be provided to the readers at separate reading sessions. Data will be recorded separately on the CRF and the readers will not have access to each other’s results.

3. Once all data has been collected and the centralised reading is closed by the sponsor, the patient details will be unblinded to allow data analysis and clinical follow-up by the PI and his team of physician-investigators as named above.

4. The investigators will read each scan in a blinded fashion, and separately. The investigators will not have access to each other’s results.

### Other methods of minimising bias

Patients will be independently randomised before the first scan is performed.

Reading for each tracers is blinded as above.

## Unblinding Procedures (Code break)

Not applicable.

# STUDY POPULATION

This single centre study will take place in the setting of a university hospital for nuclear medicine (Inselspital, Bern). The initial scan is performed in accordance with clinical routine, and is reimbursed by the patient’s insurer. Adult males with biochemically confirmed recurrent prostate cancer will be recruited. Only adults capable of informed consent and able to schedule and attend two consecutive scans are eligible.

## Eligibility criteria

*Inclusion Criteria*

- Patients with known biochemical recurrence of a histologically confirmed prostate cancer post radical prostatectomy, defined as two consecutive PSA values > 0.2 ng/ml:
- Post prostatectomy: Patients > 18 y/o
- PSA measured within ± 4 weeks of the first PSMA-PET/CT
- Patients providing written informed consent
- No change in PC treatment in the period between the first and second scans

*Exclusion Criteria*

- Patients receiving ADT within 6 months prior to the PSMA-PET/CT
- Patients with contraindication to diuresis with 20mg Furosemide
- Patients with renal dialysis or relevant renal impairment (eGFR < 35 ml/min)
- Inability to provide written informed consent
- Inability to schedule and attend two consecutive PET examinations
- Patients undergoing active treatment for a second non-prostatic malignancy at the time of the first scan.
- Known sensitivity or allergy to PSMA-ligands or one of the components of the radiotracer solutions used.

## Recruitment and screening

Patients referred for clinically routine PSMA scanning for biochemically recurrent prostate cancer are screened for eligibility by an investigator. The recruitment procedure is outlined in detail in §9.3.

## Assignment to study groups

Randomisation and allocation sequence will be performed by the trial statistician who will hold the randomisation sequence, to which the investigators will not have access.

## Criteria for withdrawal / discontinuation of participants

Patients will be withdrawn immediately from the trial if informed consent is withdrawn. Patients who die in the follow up phase will be discontinued, but not withdrawn from the trial (since follow-up to composite standard is a secondary endpoint only). Patients who, during the study, undergo active treatment for a second non-prostate malignancy will be withdrawn from secondary endpoint analysis.

### Data of withdrawn participants

Data will be handled in accordance with Art 9. ClinO [26], which we quote here in full from the relevant section:

*«Widerruft die betroffene Person ihre Einwilligung, so sind das biologische Material und die gesundheitsbezogenen Personendaten nach Abschluss der Datenauswertung zu anonymisieren».*

### Handling of Drop outs

Should a patient withdraw prior to completion of the second examination, that patient will be replaced and recruitment will continue until 100 patients have been recruited.

For patients who withdraw consent at any point, the data up to the time point of withdrawal will be used.

# STUDY INTERVENTION

## Identity of Investigational Products (treatment / medical device)

For a full description of the two radiopharmaceuticals under investigation, we refer to the “Fachinformation” (Appendix A).

### Experimental Intervention (treatment / medical device)

No experimental treatment is used. Routinely used radiopharmaceuticals are compared.

### Control Intervention (standard/routine/comparator treatment / medical device)

Intra-individual comparison of both radiopharmaceuticals, as described previously.

### Packaging, Labelling and Supply (re-supply)

Labelling is in accordance with good clinical practice and the requirements of the federal ministry of health and were approved by SwissMedic. This study is open-label in design.

### Storage Conditions

Storage of the radiopharmaceuticals is in accordance with good clinical practice.

## Administration of experimental and control interventions

### Experimental Intervention

Non applicable.

### Control Intervention

For the rationale behind our dose concept, we refer to §3.5 “Dose Rationale”. Radiation exposure to humans is as outlined in the submission to the FOPH (in parallel to this application, and included in Appendix B). For reference, we recapitulate the radiation exposure here:

Estimate of the Radiation Exposure

• ^68^Ga-PSMA-11: 150 ± 15% MBq applied activity corresponds to a maximum of 3.5 mSv for the radiopharmaceutical, with 5 mSv for the first-low dose CT (5mSv) = total 8.5 mSv

• ^18^F-PSMA-1007: 300 ± 15% MBq applied activity corresponds to 6.6 mSV for the radiopharmaceutical, with 5 mSv the first low-dose-CT = total 11.6 mSv

For the n=10 patients undergoing dynamic (parametric) acquisitions, the additional radiation dose associated with the extra low dose CT scan for attenuation correction is 1mSv, resulting in estimated radiation exposures of 9.5 and 12.6 mSv, respectively.

The additional radiation dose incurred by participation in the study (given the fact that one of the examinations is clinically indicated and is routinely performed, irrespective of whether the individual is in the study or not) is therefore below 12mSv. This is below the 20 mSv limit mandated by the FOPH.

## Dose / Device modifications

No modification of the dose or scan protocol is permitted.

## Compliance with study intervention

Directly observed administration of the radiopharmaceutical ensures compliance with the intervention.

## Data Collection and Follow-up for withdrawn participants

Patients withdrawn due to withdrawn consent will not be followed up. Data collected up until date of withdrawal will still be used. Reasons for withdrawal will be recorded on the CRF (Appendix C).

## Trial specific preventive measures

No specific preventative measures are required for this study.

## Concomitant Interventions (treatments)

No concomitant interventions are anticipated for this study.

## Study Drug / Medical Device Accountability

Accountability of these routinely used radiopharmaceuticals is in accordance with routine clinical practice. The radiopharmaceuticals as clinically routinely used radiopharmaceuticals will be taken from clinical stock and accounted for using established clinical accountability logs. Prof. Afshar-Oromieh as principal investigator is ultimately responsible for the adherence to good clinical practice.

## Return or Destruction of Study Drug / Medical Device

Surplus substance will be destroyed in accordance with routine clinical practice.

# STUDY ASSESSMENTS

## Study flow chart(s) / table of study procedures and assessments

### Study Procedures

¨


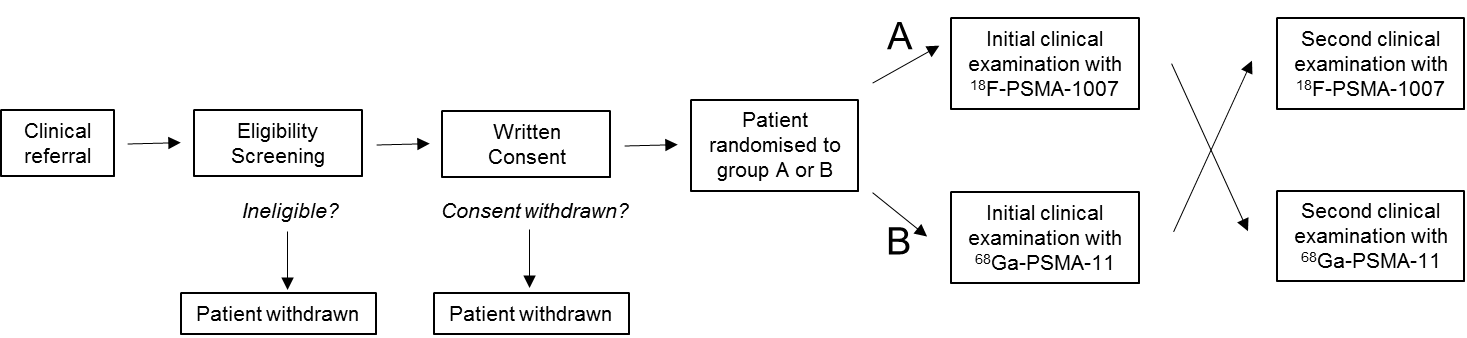


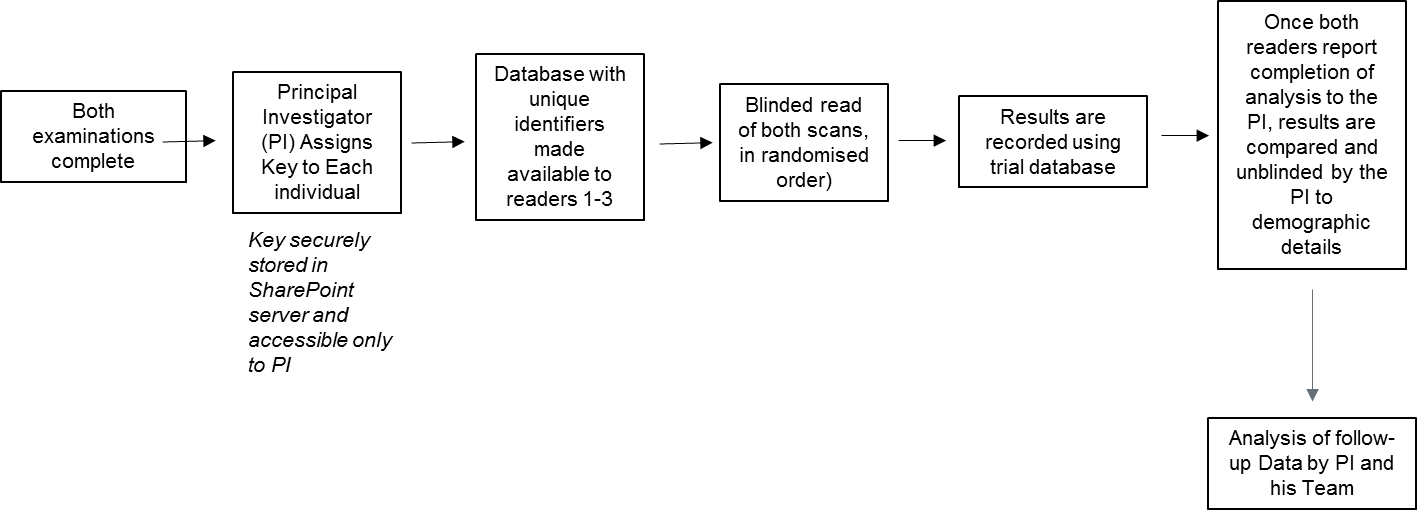


### Study Calendar

This is shown in section 1.1.1 “study calendar”

## Assessments of outcomes

The primary and secondary outcomes are listed in §5.

### Assessment of primary outcome

Six physicians will read the scans independently of each other. As outlined above in “imaging procedures” (section 6.1.2), the sponsor will provide each reader with a list of scans with a unique identifier, which allows the reader to obtain the scans in a blinded, coded and randomised fashion. The readers will be blinded to all demographic details. The sponsor will randomise the order of the scans, so that scans are not read sequentially. The readers will record the lesion radiotracer uptake and background as previously described in 6.1.2

### Assessment of secondary outcomes

Tumour to background ratio will be compared for both tracers, as described previously in this protocol.

Confirmation for lesions will only be available where the patient’s condition dictates further clinical follow up, such as repeat imaging, or where histology is performed. This data will be collected, and as a secondary explorative outcome, follow-up for lesions rated as pathological will be done by composite follow-up standard of imaging, PSA and histology up to 6 months’ follow up. It is important to note that the performance of histology, further imaging or treatment is not a study specific intervention and is performed when considered clinically appropriate by the patient’s treating physician.

For n=10 individuals parametric imaging will be performed using standard parametric imaging analysis software with only descriptive statistics.

### Assessment of other outcomes of interest

In a second round of tumour classification, two readers will classify each lesion on a lesion-specific approach in consensus (this cannot be done as a primary outcome with multiple readers, owing to the lack of a coordinate system by which multiple lesions can easily be referred to). Lesions classified as benign, equivocal or pathological will be referenced against further imaging, histology or post-treatment response to focal therapy, which will allow an exploration of the lesion-based performance of the tracers.

### Assessment of safety outcomes

Any adverse events will be recorded. Tolerability of the radiotracer will be followed up 48 hours following administration of the radiotracer by the study nurse, who will have access to a physician should any questions arise. Any AE will be assessed by a physician-investigator.

9.2.4.1 Adverse events

Any adverse event will be recorded in the individual’s clinical notes as per routine clinical usage: time of onset, duration of event, type of event, symptoms, actions taken, time to resolution, assessment of intensity. The results of any root-cause-analysis will be recorded.

Normal routine clinical procedures will be in place for the avoidance of errors in radiopharmaceutical dose and route of administration (each dose will be checked for quality in accordance with routine clinical procedures). Each dose will be double checked by two technologists for dosimetry and route of application as per clinical routine. All doses are recorded in the patient notes and the electronic radiology information system (RIS).

Reporting of Adverse events:

Reported adverse events will be recorded and classified and quantified according to the common terminology criteria for adverse events (CTCAE Version 5.0). Such data will be reported in the trial.

9.2.4.2 Laboratory parameters

Beyond those routinely provided by the referrer, no additional laboratory parameters will be obtained in this study.

9.2.4.3 Vital signs

Weight and height at the time of examination will be recorded as per clinical routine.

### Assessments in participants who prematurely stop the study

Patients who do not undergo a second scan due to an AE will be followed up as routine.

## Procedures at each visit

The study design calls for the patients to be randomised prior to the first examination. Therefore, following clinical referral, patients will be screened for eligibility. If eligible, patients are contacted and provided with the study information. If the patient agrees, following discussion with an investigator, to participate, a consent form will be provided for the patient to sign and return to us.

Patients are then randomised to group A or B and the first clinically indicated exam is scheduled. Should the patient refuse participation or withdraw his consent, the patient will receive the examination with one of the two tracers according to availability. Such a routine examination could occur with either radiotracer, both of which are routinely used both at our centre and throughout Western Europe, and both of which are approved for use in Switzerland.

The patient will have time for reflection between receiving study information and receiving the first examination (min 24hrs). A second opportunity will occur to discuss the examination prior to the second (study-specific) examination.

### Initial (clinically routine, non-study specific) Examination

The individual will be interviewed by an investigator and the patient’s formal inclusion in the study is confirmed. The first clinically routine and non-study specific examination is performed.

Patients will then be invited to return for the second examination.

### Second (study specific) Examination

The patient will return for the second examination. No further patient visits are required. The overall process is outlined in the following figure:


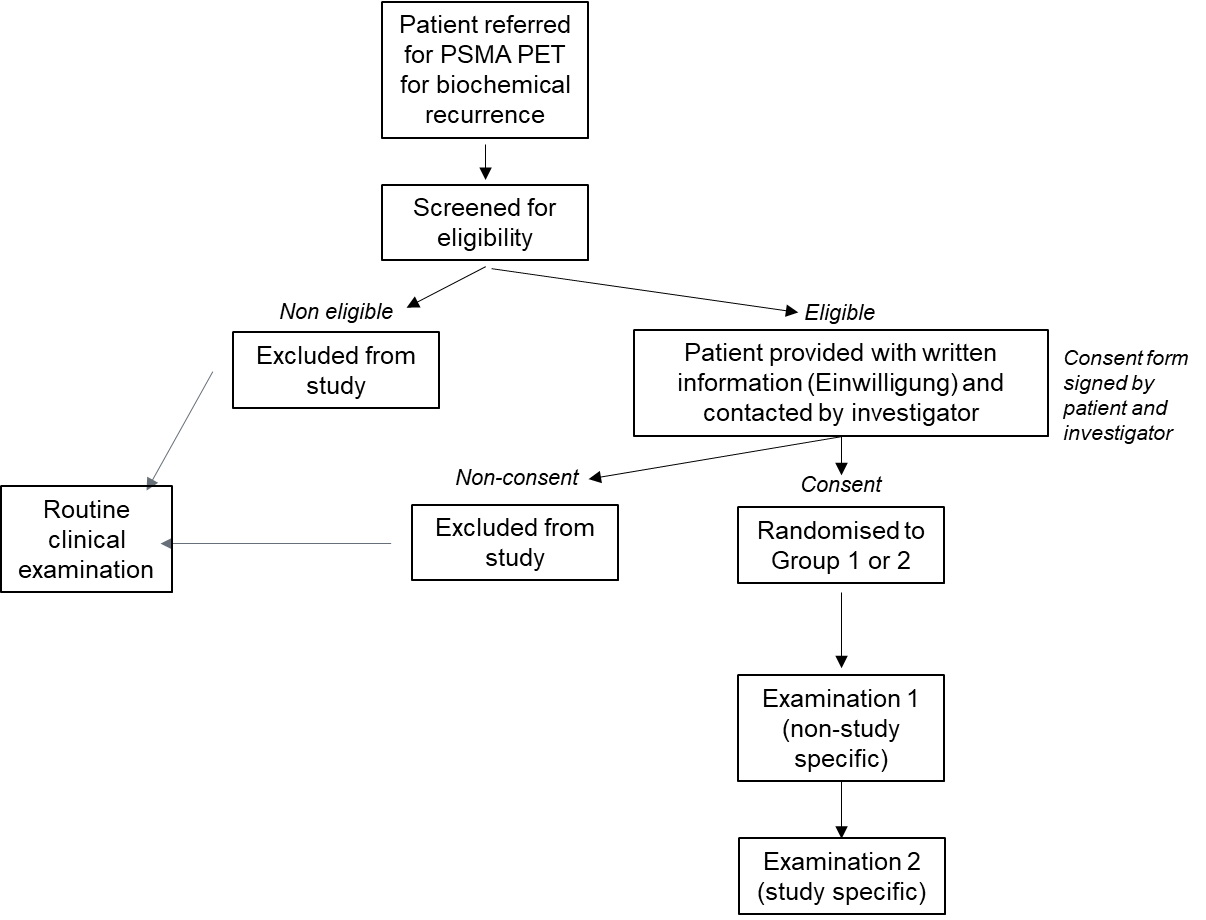


# SAFETY

## Drug studies

All adverse events (AEs) and all serious adverse events (SAEs) are collected, fully investigated and documented in source documents and case report forms (CRF) up until the follow up 48h post the second scan.

### Definition and assessment of (serious) adverse events and other safety related events

An **Adverse Event (AE)** is any untoward medical occurrence in a patient or a clinical investigation participant administered a pharmaceutical product and which does not necessarily have a causal relationship with the study procedure. An AE can therefore be any unfavourable and unintended sign (including an abnormal laboratory finding), symptom, or disease temporally associated with the use of a medicinal (investigational) product, whether or not related to the medicinal (investigational) product.

A **Serious Adverse Event (SAE)** is classified as any untoward medical occurrence that:

- results in death,
- is life-threatening,
- requires in-patient hospitalization or prolongation of existing hospitalisation,
- results in persistent or significant disability/incapacity, or
- is a congenital anomaly/birth defect.

In addition, important medical events that may not be immediately life-threatening or result in death, or require hospitalisation, but may jeopardise the patient or may require intervention to prevent one of the other outcomes listed above should also usually be considered serious.

Examples of such events are intensive treatment in an emergency room or at home for allergic bronchospasm, blood dyscrasias or convulsions that do not result in hospitalisation, or development of drug dependency or drug abuse.

SAEs should be followed until resolution or stabilisation. Participants with ongoing SAEs at study termination (including the safety visit) will be further followed up until recovery or until stabilisation of the disease after termination.

*Assessment of Causality*

Both Investigator and Sponsor make a causality assessment of the event to the study drug, based on the criteria listed in the ICH E2A guidelines:

| Relationship | Description |
| --- | --- |
| Definitely | Temporal relationship  Improvement after dechallenge*  Recurrence after rechallenge  (or other proof of drug cause) |
| Probably | Temporal relationship  Improvement after dechallenge  No other cause evident |
| Possibly | Temporal relationship  Other cause possible |
| Unlikely | Any assessable reaction that does not fulfil the above conditions |
| Not related | Causal relationship can be ruled out |
| *Improvement after dechallenge only taken into consideration if applicable to reaction | |

*Unexpected Adverse Drug Reactions*

An “unexpected” adverse drug reaction is an adverse reaction, the nature or severity of which is not consistent with the applicable product information (“Fachinformation” – Appendix A).

*Suspected Unexpected Serious Adverse Reactions (SUSARs)*

The Sponsor-Investigator evaluates any SAE that has been reported regarding seriousness, causality and expectedness. If the event is related to the investigational product and is both serious and unexpected, it is classified as a SUSAR.

*Assessment of Severity*

The severity grading scale used will be the “Common Terminology Criteria for Adverse Events CTCAE Version 5.0”.

### Reporting of serious adverse events (SAEs) and other safety related events

*Reporting of SAEs*

All SAEs must be reported immediately and within a maximum of 24 hours to the Sponsor of the study. The Sponsor will re-evaluate the SAE and return the form to the site.

SAEs resulting in death are reported to the Ethics Committee via BASEC within 7 days.

*Reporting of SUSARs*

A SUSAR needs to be reported to the Ethics Committee via BASEC and to Swissmedic for this category C study (via Sponsor) within 7 days, if the event is fatal, or within 15 days (all other events).

*Reporting of Safety Signals*

All suspected new risks and relevant new aspects of known adverse reactions that require safety-related measures, i.e. so called safety signals, must be reported to the Sponsor within 24 hours. The Sponsor must report the safety signal within 7 days to the Ethics Committee via BASEC and to Swissmedic (category C study).

*Reporting and Handling of Pregnancies*

Not applicable – only male subjects are included in this study.

*Periodic reporting of safety*

A final safety report on completion of the study will be reported to the competent authorities (CEC, Swissmedic).

An annual safety report is submitted once a year to the local Ethics Committee and to Swissmedic in this category C study via the Sponsor.

The FOPH will be furnished with a final report within one year following termination of the study regarding all aspects of radiation protection, in accordance with ClinO Art. 44.

### Follow up of (Serious) Adverse Events

Participants with ongoing SAEs at the 48h follow-up will be followed up until recovery or until stabilisation of the disease after termination. Vital signs, any laboratory values obtained and the clinical condition of the patient will be recorded in the patient’s clinical notes. In cases of loss to follow up, the patient’s general practitioner will be contacted.

## Medical Device Category C studies

Section does not apply.

## Medical Device Category A studies

Section does not apply.

## Assessment, notification and reporting on the use of radiation sources

Under consideration of the potential diagnostic benefit to be gained from examination with the additional radiopharmaceutical [27], and in accordance with article 45 of the Radiological Protection Ordinance of 26 April 2017 (subsection 2), the effective dose incurred by participation in this trial falls below 20mSv.

If the permitted dose guidance value is exceeded at any time, the investigator notifies the Ethics Committee via BASEC within 7 working days of it becoming known.

In this category C clinical trial, a diagnostic radiopharmaceutical is used. No therapeutic products that emit ionising radiation will be used.

# STATISTICAL METHODS

## Hypothesis

**Step 1: Hypotheses**

Null Hypothesis –The new tracer is inferior to the old tracer with respect to the detection of patients with pathological PSMA-positive findings using a non-inferiority margin of -10%, i.e.

Pr(PSMA+)new – Pr(PSMA+)old < -10%

Alternative Hypothesis – The new tracer is non-inferior to the old tracer with respect to the detection of patients with pathological PSMA-positive findings using a non-inferiority margin of -10%, i.e.

Pr(PSMA+)new – Pr(PSMA+)old > -10%

## Determination of Sample Size

Power was determined using Monte Carlo simulations with 1000 repetitions and the following assumptions:

- Marginal probabilities: 80% of the patients are positive for tracer A and 80% are positive for tracer B
- Both tracers are applied to each patient
- Non-inferiority design with a one-sided alpha of 2.5%
- Non-inferiority margin of -10% (clinically acceptable margin in detection rate, balanced against the requirements for clinically realistic number of patients). We justify this margin as being less than 50% of the margin reported for superiority (22%) in a prospective trial published for ^68^Ga-PSMA-11 versus the first-generation tracer ^18^F-Choline [22].
- Non-inferiority is concluded if the lower limit of a 95% confidence interval (Wald with Bonett–Price Laplace adjustment) for the difference in the proportion of tracer-positive patients (new tracer – old tracer) is larger than the non-inferiority margin
- A pragmatic assumption is made regarding the correlation of the paired measurements. Limited data are available for this, given the dearth of comparative studies. Neither retrospective analyses [28] nor limited pilot studies [17, 27] have demonstrated any difference in detection rate. We take a worst-case scenario, and assume that both tracers are positive for 75% of the patients.

Data was simulated using function rmvbin from R package bindata [29]. With 100 patients we will have a power of 85% to detect non-inferiority.

## Statistical criteria of termination of trial

No statistical criteria are planned.

## Planned Analyses

A detailed description of data preparation and analysis will be elaborated as below:

### Datasets to be analysed, analysis populations

The full analysis set will include all randomised patients. They will be analysed according to the group they were randomised to regardless of the tracer actually used according to the intention-to-treat principle. We will also define a per-protocol analysis set that excludes major protocol violations.

### Primary Analysis

The primary outcome (proportion of patients with PSMA-positive pathological lesions) will be compared between tracers using an absolute risk difference (new tracer minus old tracer) with a one-sided lower 95% confidence interval (CI) for paired proportions (Wald with Bonett–Price Laplace adjustment). If the lower limit lies above -10%, we will claim non-inferiority. Proportion of pathologic scans will be based on majority consensus reads.

The number of PSMA-positive lesions defined as pathological, benign and uncertain in each region will be analysed using separate mixed-effects Poisson regression models with tracer as fixed covariate and nested random effects for patient and region. We will report incidence rate ratios with 95% CI and p-values. As a sensitivity analysis we will compare the number over all regions using the Wilcoxon signed-rank tests and the Mann-Whitney statistic with 95% CI.

Inter-reader reliability will be analysed on the region-level based on the number of benign, pathological and uncertain lesions determined by each reader. We will calculate Krippendorff’s alpha with a ratio difference function and bootstrap 95% CI separately for each tracer.

Lesion-based PPV will be calculated for each tracer as the proportion of pathological lesions confirmed pathological during follow-up among all lesions with a known state and will be presented with 95% Wilson score CI. Using all lesions with a confirmed status during follow-up, the two tracers will be compared using McNemar’s test and an absolute risk difference with 95% confidence interval.

The semi-quantitative tumour to background ratio will be summarized on a region-level (i.e. using the mean over all positive lesions per region) and analysed using a linear mixed-effects regression model with tracer as fixed covariate and patient and region as nested random effects. The mean difference with 95% CI will be reported.

For the subset of patients with additional parametric imaging data (N=10) the planned analysis of parametric imaging parameters (see first secondary endpoint) is planned prior to completion of the study. This exploratory analysis of this secondary endpoint will not assess the primary endpoint and will not influence further trial conduct or further trial process.

### Secondary Analyses

Secondary analyses of all outcomes will be done in the per-protocol analysis set.

### Subgroup analyses

No subgroup analyses are planned.

### Interim analyses

None planned.

### Safety analysis

The number and severity of adverse events (up to 48h post exam) will be summarised by tracer using descriptive statistics. All serious adverse events will be listed.

### Deviation(s) from the original statistical plan

Deviations from the original statistical analysis plan will be requested in writing by the PI to the trial statistician, and approved by the project sponsor.

## Handling of missing data and drop-outs

For patients lost to follow up, all reasonable measures to contact the patient or his treating physician will be taken.

For patients who withdraw consent at any point, the data up to the time point of withdrawal will be used.

# QUALITY ASSURANCE AND CONTROL

The PI has ultimate responsibility for adherence to quality control and quality assurance procedures.

## Data handling and record keeping / archiving

### Case Report Forms

Data is entered directly into an electronic Case Report Form (e-CRF). For each enrolled study participant an eCRF is maintained. eCRFs must be kept current to reflect subject status at each phase during the course of study. Participants will not be identified in the eCRF by name or initials and birth date. Appropriate coded identification by means of a unique participant number must be used. The principal investigator will maintain this list in the investigator site file and is only accessible to study team members but will be made available as appropriate during monitoring visits, audits and site inspections.

The eCRF will be made available to authorised personnel via the REDCap system. Only those authorised will have access to the eCRF. No investigator has access to another investigator’s eCRF. The Sponsor has access to all eCRFs.

### Specification of source documents

Source data will be available at the site to document the study participants. Source data includes all original documents relating to the study, as well as the medical treatment and medical history of the participant (clinical notes).

Demographic data, examination dates, participation in the study:

Demographic data, visit dates, participation in the study and Informed Consent Forms, randomisation number, SAEs, AEs and concomitant medication, results of relevant examinations and results of any clinical follow up are all recorded on the eCRF.

All data that are directly recorded in the eCRF are to be considered source data. All source data pertaining to an individual will be electronically scanned and uploaded to the patient’s notes which are held in the RIS (Radiological Information System) of the Inselspital, including consent forms. The original paper forms will be held in the trial master file.

### Record keeping / archiving

All study data must be archived for a minimum of ten years after study termination or premature termination of the clinical trial. Electronic storage is as outlined in 12.2

## Data management

Data will be captured using the REDCap system. Scientific staff will have access to the data in codified form.

### Data Management System

Data obtained in this study will be managed by the REDCap system which is provided by the Clinical Trials Unit (CTU) Bern and is HRA and GCP compliant.

### Data security, access and back-up

Details of the Clinical Trials Unit Bern’s data security, access and back-up procedures are available on their website [https://www.ctu.unibe.ch/services/data_management/index_eng.html]. In summary: CTU Bern provides and maintains secure and up-to-date IT infrastructure. The servers that host the study databases are housed in dedicated server facilities at the University of Bern or at the Inselspital Bern. Together with their IT department, they update the CDMS as well as all other software on the servers. They regularly back up study- and meta-data, according to a detailed back-up plan. Internal back-ups are made several times per day, and daily back-ups are stored on external disks.

Their security measures meet the standards of the “Datenschutzaufsichtsstelle des Kantons Bern”.

### Analysis and archiving

Data will be extracted by the physician reading the scan independently and uploading the data into the CRF, which will be individually available to each reader. The PI will collate all CRFs at the conclusion of the study.

### Electronic and central data validation

Data ranges will be monitored. Each scan will be read by multiple independent physicians, ensuring an internal validation of the data.

Data will be recorded using the REDCap database as provided by the Clinical Trials Unit. The investigator will be responsible for data added to the database. He or she will confirm this by signature.

## Monitoring

The monitoring plan uses a risk-based approach as published by the Swiss Clinical Trials Organisation (SCTO). Monitoring will be carried out by a monitoring team from the Clinical Trials Unit (CTU) Bern. The final monitoring plan is attached as an annex to this protocol.

## Audits and Inspections

No separate audit is planned.

## Confidentiality, Data Protection

Direct access to source documents will be permitted for purposes of monitoring (12.3), audits and inspections (12.4) (ICH E6, 6.10). The PI will retain access to the protocol and dataset during and after the study. Following publication of the study, coded data-sets may be provided at the discretion of the sponsor for the purposes of scientific peer-review.

## Storage of biological material and related health data

No biological material stored. Storage of data is as per §12.2.2.

# PUBLICATION AND DISSEMINATION POLICY

The trial results will be published in a peer reviewed journal and disseminated via national and international scientific congresses.

# FUNDING AND SUPPORT

## Funding

This trial is supported by grants from the Berger-Janser Stiftung zur Krebserforschung, Gesuch Nr 11/2019 and the bernische Krebsliga. A CTU grant from the Direktion Lehre und Forschung, Inselspital Bern to cover the personnel costs of Dr. I.L. Alberts has been awarded.. No other grants have been submitted or are anticipated.

## Other Support

The radiopharmaceuticals are kindly provided by Unilabor Bern and SwanIsotopen AG.

# INSURANCE

Once registered with the Department Lehre und Forschung, Inselspital, the trial will come under the general trial insurance of the Inselspital. A written confirmation will be kept in the trial master file.

# REFERENCES

1. Afshar-Oromieh A, Hetzheim H, Kubler W, Kratochwil C, Giesel FL, Hope TA, et al. Radiation dosimetry of (68)Ga-PSMA-11 (HBED-CC) and preliminary evaluation of optimal imaging timing. European journal of nuclear medicine and molecular imaging. 2016;43:1611-20. doi:10.1007/s00259-016-3419-0.

2. Heilmittelgesetz (HMG; SR 812.21), Artikel 9, Absatz 2, Buchstabe a.

3. Arzneimittelverordnung (VAM; SR 812.212.21), Artikel 37, Absatz 1, Buchstabe e, which references to «Anhang 1» of the same ordinance.

4. Siegel RL, Miller KD, Jemal A. Cancer statistics, 2018. CA Cancer J Clin. 2018;68:7-30. doi:10.3322/caac.21442.

5. Afshar-Oromieh A, Holland-Letz T, Giesel FL, Kratochwil C, Mier W, Haufe S, et al. Diagnostic performance of 68Ga-PSMA-11 (HBED-CC) PET/CT in patients with recurrent prostate cancer: evaluation in 1007 patients. European journal of nuclear medicine and molecular imaging. 2017;44:1258-68. doi:10.1007/s00259-017-3711-7.

6. Fech V, Alberts I, Rominger A, Afshar-Oromieh A. ﻿PSMA-ligand PET allows a more accurate therapeutic response evaluation of bone metastases in prostate cancer compared to computed tomography. Nuklearmedizin. doi:10.1055/a-0895-5078.

7. Alberts I, Sachpekidis C, Gourni E, Boxler S, Gross T, Thalmann G, et al. Dynamic patterns of [(68)Ga]Ga-PSMA-11 uptake in recurrent prostate cancer lesions. Eur J Nucl Med Mol Imaging. 2019. doi:10.1007/s00259-019-04545-8.

8. Zschaeck S, Lohaus F, Beck M, Habl G, Kroeze S, Zamboglou C, et al. PSMA-PET based radiotherapy: a review of initial experiences, survey on current practice and future perspectives. Radiation oncology. 2018;13:90. doi:10.1186/s13014-018-1047-5.

9. Onal C, Torun N, Akyol F, Guler OC, Hurmuz P, Yildirim BA, et al. Integration of 68Ga-PSMA-PET/CT in Radiotherapy Planning for Prostate Cancer Patients. Clinical nuclear medicine. 2019. doi:10.1097/RLU.0000000000002691.

10. Fossati N, Karnes RJ, Colicchia M, Boorjian SA, Bossi A, Seisen T, et al. Impact of Early Salvage Radiation Therapy in Patients with Persistently Elevated or Rising Prostate-specific Antigen After Radical Prostatectomy. European urology. 2017. doi:10.1016/j.eururo.2017.07.026.

11. Israeli RS, Powell CT, Corr JG, Fair WR, Heston WDW. Expression of the Prostate-specific Membrane Antigen. Cancer Research. 1994;54:1807.

12. Afshar-Oromieh A, Zechmann CM, Malcher A, Eder M, Eisenhut M, Linhart HG, et al. Comparison of PET imaging with a (68)Ga-labelled PSMA ligand and (18)F-choline-based PET/CT for the diagnosis of recurrent prostate cancer. European journal of nuclear medicine and molecular imaging. 2014;41:11-20. doi:10.1007/s00259-013-2525-5.

13. Eiber M, Maurer T, Souvatzoglou M, Beer AJ, Ruffani A, Haller B, et al. Evaluation of Hybrid (6)(8)Ga-PSMA Ligand PET/CT in 248 Patients with Biochemical Recurrence After Radical Prostatectomy. Journal of nuclear medicine : official publication, Society of Nuclear Medicine. 2015;56:668-74. doi:10.2967/jnumed.115.154153.

14. Afshar-Oromieh A, Avtzi E, Giesel FL, Holland-Letz T, Linhart HG, Eder M, et al. The diagnostic value of PET/CT imaging with the (68)Ga-labelled PSMA ligand HBED-CC in the diagnosis of recurrent prostate cancer. European journal of nuclear medicine and molecular imaging. 2015;42:197-209. doi:10.1007/s00259-014-2949-6.

15. Virgolini I, Decristoforo C, Haug A, Fanti S, Uprimny C. Current status of theranostics in prostate cancer. European journal of nuclear medicine and molecular imaging. 2018;45:471-95. doi:10.1007/s00259-017-3882-2.

16. Sachpekidis C, Kopka K, Eder M, Hadaschik BA, Freitag MT, Pan L, et al. 68Ga-PSMA-11 Dynamic PET/CT Imaging in Primary Prostate Cancer. Clinical Nuclear Medicine. 2016;41.

17. Kuten J, Fahoum I, Savin Z, Shamni O, Gitstein G, Hershkovitz D, et al. Head- to head Comparison of (68)Ga-PSMA-11 with (18)F-PSMA-1007 PET/CT in Staging Prostate Cancer Using Histopathology and Immunohistochemical Analysis as Reference-Standard. Journal of nuclear medicine : official publication, Society of Nuclear Medicine. 2019. doi:10.2967/jnumed.119.234187.

18. Alberts I, Sachpekidis C, Dijkstra L, Prenosil G, Gourni E, Boxler S, et al. The role of additional late PSMA-ligand PET/CT in the differentiation between lymph node metastases and ganglia. European journal of nuclear medicine and molecular imaging. 2020;47:642-51. doi:10.1007/s00259-019-04552-9.

19. Haupt F, Dijkstra L, Alberts I, Sachpekidis C, Fech V, Boxler S, et al. (68)Ga-PSMA-11 PET/CT in patients with recurrent prostate cancer-a modified protocol compared with the common protocol. Eur J Nucl Med Mol Imaging. 2019. doi:10.1007/s00259-019-04548-5.

20. Fendler WP, Calais J, Eiber M, Flavell RR, Mishoe A, Feng FY, et al. Assessment of 68Ga-PSMA-11 PET Accuracy in Localizing Recurrent Prostate Cancer: A Prospective Single-Arm Clinical Trial. JAMA Oncology. 2019;5:856-63. doi:10.1001/jamaoncol.2019.0096.

21. Rowe SP, Pienta KJ, Pomper MG, Gorin MA. PSMA-RADS Version 1.0: A Step Towards Standardizing the Interpretation and Reporting of PSMA-targeted PET Imaging Studies. European urology. 2018;73:485-7. doi:10.1016/j.eururo.2017.10.027.

22. Calais J, Ceci F, Eiber M, Hope TA, Hofman MS, Rischpler C, et al. 18F-fluciclovine PET-CT and 68Ga-PSMA-11 PET-CT in patients with early biochemical recurrence after prostatectomy: a prospective, single-centre, single-arm, comparative imaging trial. The Lancet Oncology. 2019;20:1286-94. doi:<https://doi.org/10.1016/S1470-2045(19)30415-2>.

23. Scher HI, Halabi S, Tannock I, Morris M, Sternberg CN, Carducci MA, et al. Design and end points of clinical trials for patients with progressive prostate cancer and castrate levels of testosterone: recommendations of the Prostate Cancer Clinical Trials Working Group. Journal of clinical oncology : official journal of the American Society of Clinical Oncology. 2008;26:1148-59. doi:10.1200/JCO.2007.12.4487.

24. <https://jamanetwork.com/journals/jamaoncology/fullarticle/2729065>.

25. Emmett L, Metser U, Bauman G, Hicks RJ, Weickhardt A, Davis ID, et al. Prospective, Multisite, International Comparison of 18F-Fluoromethylcholine PET/CT, Multiparametric MRI, and 68Ga-HBED-CC PSMA-11 PET/CT in Men with High-Risk Features and Biochemical Failure After Radical Prostatectomy: Clinical Performance and Patient Outcomes. Journal of Nuclear Medicine. 2019;60:794-800.

26. 810.305 Verordnung über klinische Versuche in der Humanforschung (Verordnung über klinische Versuche; KlinV).

27. Dietlein F, Kobe C, Hohberg M, Zlatopolskiy BD, Krapf P, Endepols H, et al. Intraindividual comparison of 18F-PSMA-1007 with renally excreted PSMA ligands for PSMA-PET imaging in patients with relapsed prostate cancer. Journal of Nuclear Medicine. 2019.

28. Rauscher I, Kronke M, Konig M, Gafita A, Maurer T, Horn T, et al. Matched-pair comparison of (68)Ga-PSMA-11 and (18)F-PSMA-1007 PET/CT: frequency of pitfalls and detection efficacy in biochemical recurrence after radical prostatectomy. J Nucl Med. 2019. doi:10.2967/jnumed.119.229187.

29. <https://cran.r-project.org/web/packages/bindata/bindata.pdf>.

# APPENDICES

A Fachinformation – Product Information Leaflet for the two radiotracers

B FOPH Application Copy

C Draft CRF- Case Report Form
